# Molecular Design Toward High‐Performance Solution‐Processable Push–Pull Zinc(II) Porphyrin‐Based Resistive Memory Devices: From Binary to Ternary Memory Behavior

**DOI:** 10.1002/smsc.70285

**Published:** 2026-05-15

**Authors:** Ka Wai Kwong, Hing Chan, Ming‐Yi Leung, Eugene Yau‐Hin Hong, Shiu‐Lun Lai, Vivian Wing‐Wah Yam

**Affiliations:** ^1^ Department of Chemistry The University of Hong Kong Hong Kong P. R. China; ^2^ Hong Kong Quantum AI Lab Limited Hong Kong P. R. China

## Abstract

A series of zinc(II) porphyrin‐based donor−acceptor (D–A) decorated complexes has been designed, synthesized, characterized, and employed to fabricate solution‐processable resistive memory devices. High‐performance ternary memory devices based on these complexes have been demonstrated by the well‐separated current ratios of 1:10^3^:10^6^ with the “OFF”, “ON1”, and “ON2” resistive states. A long retention time of over 20,000 s has also been achieved. Instead of using strong electron‐donating and withdrawing moieties, this work has demonstrated the utilization of weak electron‐donating and withdrawing units to realize ternary memory behaviors. Together with the photophysical, electrochemical, and computational studies, ternary memory behaviors have been assigned as originating from the charge‐trapping state of the porphyrin core and charge transfer processes associated with the D–A moieties. The present study has demonstrated the structure–property relationship of the memory devices and offers important insights as well as design strategies for the development of multilevel organic resistive memory devices.

## Graphical abstract

A series of zinc(II) porphyrin‐based D−A complexes is employed to fabricate solution‐processable resistive memory devices. The utilization of an electron‐donating π‐bridge or a weak electron‐donating moiety is able to turn on an extra resistive state to realize ternary memory performance, demonstrating the relationship between memory behavior and molecular structure.

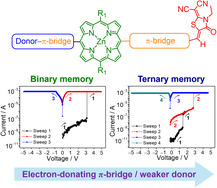

## 1 Introduction

Over the past few decades, memory devices such as SRAM (Static Random‐Access Memory), DRAM (Dynamic Random‐Access Memory), and SSDs (Solid‐State Drives) have become essential components in electronic devices. These devices serve as the primary means for storing and processing data, enabling the efficient and reliable operation of modern electronic systems [1, 2]. The advancement of new memory architectures and fabrication processes, such as 3D NAND flash memory and FinFET technology, has enabled the production of memory devices with greater capacity in smaller form factors [3, 4, 5]. On the other hand, the development of memory devices based on alternative memory mechanisms, such as phase‐change memory, ferroelectric memory, magnetic random access memory, and resistive random access memory (RRAM) [6, 7], has attracted increasing attention due to their potential to achieve higher areal density, lower power consumption, and faster read/write speeds, making them attractive options for future memory devices [4, 5, 6].

Among the alternative memory mechanisms, RRAM has shown particular promise due to its simple device structure, low production cost, and low power consumption [5, 8, 9, 10, 11]. RRAM devices consist of a thin metal oxide film sandwiched between two electrodes, and data are stored by changing the resistance of the memory materials through the application of an electric field. These design strategies offer several advantages compared to other inorganic or polymer‐based devices, such as molecular level tunability and solution‐processability for the realization of low‐power, high‐density, and multilevel storage [5, 8, 9, 10, 11]. The resistance of the memory material layer can be changed by applying an electric field across the device. This results in a change in the resistance of the device, with the high and low resistive states representing the “OFF” and “ON” states, respectively. Such a switching behavior between different resistive states enables resistive memory devices to store data, making them attractive for applications requiring high‐density data storage [12, 13, 14, 15, 16]. As a result, significant research efforts are underway to optimize the performance and reliability of resistive memory devices for commercial use. In general, most memory materials exhibit binary memory behavior, representing two different states, “0” and “1”, for use in the coding system [4]. More recently, various organic and organometallic materials have demonstrated ternary and quaternary memory behaviors, in which the density of data storage can be significantly increased from binary (2^*n*^) to ternary (3^*n*^) or quaternary (4^*n*^) [9, 12, 13, 14, 15, 16, 17, 18, 19, 20, 21, 22, 23, 24, 25, 26, 27]. For example, organogold(III) complexes with electron‐withdrawing phosphole oxide were demonstrated to exhibit ternary memory behavior [23]. Multilevel memory behaviors of boron(III) containing donor−acceptor (D–A) compounds have also been studied, and it is suggested that stepwise charge transfer processes are important for achieving ternary memory behavior [24]. Moreover, ternary memory behaviors have also been realized in axially substituted subphthalocyanine boron(III) compound, indicating that the well‐separated charge‐transfer state is effective to achieve multilevel memory behaviors [26]. Furthermore, the use of different strong acceptors on a single molecule was reported to show multilevel resistive memory behavior [9, 17, 18, 19]. In 2016, a quaternary resistive memory device based on D–A molecules was reported that utilized multiple strong electron acceptors, suggesting that the quaternary memory behavior is originated from field‐induced charge‐transfer processes [20]. These examples have demonstrated that multilevel memory behavior can be achieved by molecular engineering. However, the golden rule for the precise design of multilevel resistive memory devices is yet to be defined, and more efforts to unveil the relationship between the molecular structure and memory behavior are needed.

Porphyrin is a family of compounds that has been extensively researched due to its unique properties and ease of synthesis, making it a promising candidate for various functional materials [28, 29, 30, 31, 32]. In particular, porphyrin‐based dye‐sensitized solar cells with donor−acceptor dyad, donor−π−acceptor (D−π−A), and other triad structures have demonstrated high power conversion efficiency and tunable charge‐transfer properties [33, 34, 35, 36]. The solution‐processable resistive memory devices based on push–pull *β*‐substituted nickel(II) porphyrin were reported in 2017, where ternary memory behaviors have been realized [37]. This study suggested that the two low‐resistive states, “ON1” and “ON2”,originate from the charge‐transfer process from the *β*‐pyrrolic substituent to the porphyrin core and charge‐trapping state of the nickel(II) porphyrin core, respectively. More importantly, this study has demonstrated that the utilization of weak electron‐donating and withdrawing functional groups at the porphyrin core could induce ternary memory behavior [37], which is different from the recent report that strong electron‐donating or withdrawing moieties are critical for multilevel memory behaviors [20, 38]. Recently, a series of *meso*‐substituted porphyrin compounds was reported to show organic resistive memory behaviors. These devices showed tunable switching threshold voltages (*V*_th_) of −2.32 and −2.97 V through the planarization and extension of conjugation by the incorporation of alkynyl π‐bridges between porphyrin units and anhydride moieties [39]. However, multilevel memory behaviors are not realized in these types of compounds. Nevertheless, the ease of synthesis and well‐explored photophysical and electrochemical properties of porphyrin render them excellent candidates for the investigation of the structure–property relationship required for multilevel resistive memory materials.

**FIGURE 1:**
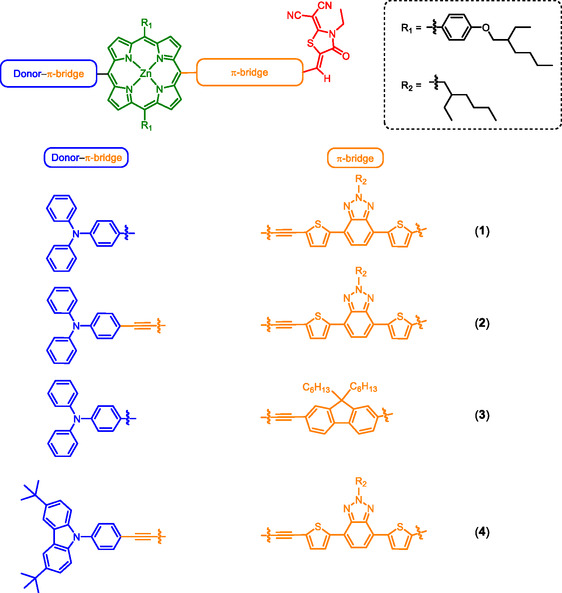
Molecular structures of complexes **1**−**4**. Donor (blue), Por (green), π‐bridge (orange), acceptor (red).

Herein, a series of zinc(II) porphyrin‐based D−A complexes has been designed, synthesized, and characterized (Figure 1). Zinc(II) porphyrin (Por) is employed as the core unit and is combined with different donors and π‐bridges, while keeping the acceptor moiety unchanged, to study the relationship between resistive memory behavior and molecular structure. Detailed photophysical and electrochemical studies have been performed to investigate the electronic properties of these complexes. Solution‐processed resistive memory devices based on these complexes have been fabricated and are found to exhibit binary or ternary memory behaviors, dependent on the choice of the donor and π‐bridge moieties, suggesting their important role in the realization of ternary memory performance. This study demonstrates the potential of porphyrin‐based materials as a promising candidate for resistive memory applications, and more importantly, provides insights into achieving multilevel memory behavior by molecular design.

## 2 Results and Discussion

### 2.1 Synthesis and Characterizations

D−Por−π (precursors of **1** and **3**) and D−π−Por−π (precursors of **2** and **4**) were prepared through Sonogashira coupling reactions between the corresponding π‐bridge and porphyrin moieties, while the π‐bridge was synthesized according to the previously reported methods [14, 24, 25]. Strong electron‐withdrawing acceptor moiety of (1,1‐dicyanomethylene)‐3‐octyl rhodanine (CNR) was prepared by the literature‐reported procedure [28]. The final complexes were synthesized by Knoevenagel condensation between (1,1‐dicyanomethylene)‐3‐octyl rhodanine (CNR) acceptor and the aldehyde unit of the π‐bridge. The synthetic pathways of complexes **1**−**4** are summarized in Schemes S1−S4. 2‐Ethylhexyl and hexyl chains of the porphyrin core and π‐bridges are used to improve the solubility of the complexes to increase their processability for solution‐processing methods. All the complexes are found to be air‐stable and have been characterized by nuclear magnetic resonance spectroscopy, high‐resolution electrospray ionization mass spectrometry, and elemental analyses.

### 2.2 Photophysical Properties

**FIGURE 2:**
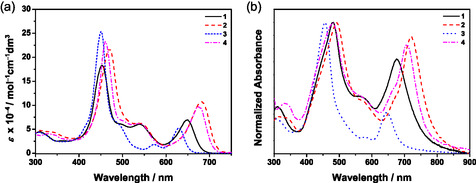
Electronic absorption spectra of **1**−**4** (a) in toluene solution and (b) in neat film at 298 K.

**FIGURE 3:**
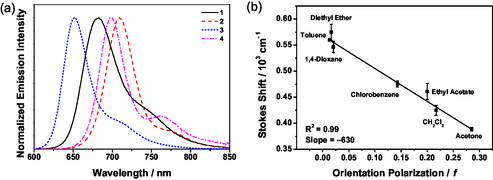
(a) Emission spectra of **1**−**4** in degassed toluene solution. (b) Lippert−Mataga plot of **2** at 298 K.

The electronic absorption spectra of **1**−**4** in toluene solution display intense Soret bands at *ca.* 451−470 nm with extinction coefficients (ε) in the order of 10^5^ dm^3^mol^−1^cm^−1^, and Q‐bands at *ca.* 573−681 nm with ε in the order of 10^4^ dm^3^mol^−1^cm^−1^ (Figure 2a and Table S1). Complexes **1**−**2** and **4** display similar absorption bands at 538−540 nm, which are assigned to the intraligand (IL) transition of the thiophene‐benzotriazole π‐bridge with mixing of an intramolecular charge transfer (ICT) transition from the thiophene to the terminal acceptors of the π‐bridge. The incorporation of the alkynyl moiety between the donor and porphyrin in **2** results in a red‐shifted Soret and Q‐bands when compared to that of **1**, suggesting an increase in the ICT character from the donor to acceptor upon planarization of the donor and porphyrin moieties [28, 40]. Meanwhile, complexes **2** and **4** displayed Soret bands at *ca*. 470 and 461 nm and Q‐bands at *ca.* 681 and 675 nm, respectively, indicating that the replacement of diphenylamine by the *t*‐butylcarbazole moiety would weaken the ICT [36, 41]. Moreover, complex **3** exhibits two Q‐bands at 573 nm and 629 nm, while **1**−**2** and **4** display only one Q‐band. It is believed that the electron‐donating 9,9‐dihexylfluorene π‐bridge may weaken the ICT in **3** [41]. The Q‐band intensity for **3** is also found to be lower than that of **1**. In contrast to **3** with a relatively electron‐donating π‐bridge, **1** features a more electron‐withdrawing π‐bridge that can impart a larger extinction coefficient for the Q‐band transition. In addition, there is a red shift of the Q‐band of **4** relative to **1**, which can be attributed to the alkynyl π‐bridge in **4** that facilitates the planarization between the donor moiety (*t*‐butylcarbazole) and the porphyrin core. This leads to a more conjugated structure and enhanced charge transfer, even though a weaker *t*‐butylcarbazole donor is present in **4**. A similar trend is also observed upon comparing the Q‐band of **1** and **2**. Furthermore, the electronic absorption spectra of **1**−**4** in solid‐state thin film have also been investigated (Figure 2b). In general, thin films of **1**−**4** display broadened and red‐shifted absorptions when compared to their corresponding absorption spectra in toluene solution, possibly due to the presence of strong intermolecular π−π interactions in the neat film [28, 40]. In particular, the intensities of the Q‐bands of complexes **1**, **2**, and **4** relative to the Soret bands show a drastic increase, indicating the addition of alkynyl moieties could planarize the molecular structure and induce stronger intermolecular π−π interactions in the solid‐state thin films. However, complex **3** shows a similar intensity ratio of Soret band and Q‐bands in both toluene solution and neat film, implying only weak intermolecular π−π interactions are present (Figure S1). Such observations are in line with the stronger intermolecular π−π stacking in the neat film arising from the planarization of the thiophene‐benzotriazole‐based π‐bridge, and of the much reduced aggregation with the introduction of the sterically bulky 9,9‐dihexylfluorene moiety. Upon excitation at *λ* > 470 nm, all the complexes are found to be emissive in degassed toluene solution at 298 K and display Gaussian‐shaped emission bands. The emission spectra of **1**−**4** are shown in Figure 3a, and the emission data are summarized in Table S2. **1** shows an emission profile with a maximum at *ca.* 683 nm, while with the addition of the alkynyl group, **2** displays a red‐shifted emission band at *ca.* 708 nm. The replacement of electron‐withdrawing thiophene‐benzotriazole‐based π‐bridge by electron‐donating 9,9‐dihexylfluorene in **3** leads to a blue‐shifted emission band at *ca.* 652 nm. A blue shift to *ca*. 698 nm is also observed by replacing the diphenylamine in **2** with *t*‐butylcarbazole in **4**. Solvent‐dependent studies have also been performed on **2** (Figures S2, S3). The Lippert−Mataga plot is shown in Figure 3b with a slope found to be −630 cm^−1^, suggesting a greater stabilization of the ground state than the excited state upon increasing solvent polarity. Therefore, these emissions are suggested to be originated from IL [π → π*(Por)] excited states with some mixing of charge transfer characters from the donor to acceptor.

### 2.3 Electrochemical Properties

**FIGURE 4:**
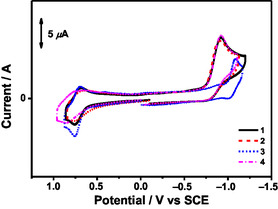
Cyclic voltammograms of complexes **1**−**4** in dichloromethane (0.1 M ^*n*^Bu_4_NPF_6_). Scan rate = 100 mV s^−1^.

The electrochemical properties of complexes **1**−**4** in dichloromethane (0.1 M ^*n*^Bu_4_NPF_6_) have been investigated by cyclic voltammetry (Figure 4 and Table 1). Complexes **1** and **3** display quasi‐reversible oxidation couples at +0.72 and +0.73 V versus saturated calomel electrode (SCE), respectively. Meanwhile, complexes **2** and **4** exhibit irreversible oxidation waves at +0.76 and +0.86 V versus SCE, respectively. These oxidative waves are found to be sensitive to the nature of the donor, and therefore can be assigned as originating from the oxidation of the donor moieties. For the reductive scan, all the complexes show an irreversible reduction wave at −0.91 to −1.09 V versus SCE. Complexes **1**, **2**, and **4** with the same π‐bridge and acceptor show a similar reduction wave of −0.91 to −0.92 V versus SCE, while complex **3** with an electron‐donating π‐bridge displays a more negative reduction wave at −1.09 V versus SCE. These reduction waves are assigned as originating from the reduction of the π‐bridge and acceptor. Similar assignments have also been reported in related porphyrin complexes [28, 43].

**TABLE 1:** Electrochemical data for complexes **1**−**4**.^a^

Complexes	Oxidation, *E*_1/2_, V. vs. SCE,^b^ (Δ*E*_p_/mV) [*E*_pa_, V. vs. SCE^c^]	Reduction, *E*_1/2_, V. vs. SCE^b^ [E_pc_, V. vs. SCE^d^]	Calculated^e^ HOMO/eV	Calculated^f^ LUMO/eV
**1**	+0.72 (71)	[−0.91]	−5.06	−3.43
**2**	[+0.76]	[−0.92]	−5.09	−3.42
**3**	+0.73 (67)	[−1.09]	−5.07	−3.25
**4**	[+0.86]	[−0.92]	−5.20	−3.42

a In dichloromethane solution with ^*n*^Bu_4_NPF_6_ (0.1 M) as the supporting electrolyte at room temperature; scan rate 100 mVs^−1^.

b Quasi‐reversible oxidation couple, *E*_1/2_ = (*E*_pa_ + *E*_pc_)/2; *E*_pa_ and *E*_pc_ are peak anodic and peak cathodic potentials, respectively. The values in the brackets refer to the difference between anodic peak and cathodic peak potentials, Δ*E*_p_ = |*E*_pa_ − *E*_pc_|.

c *E*_pa_ refers to the anodic peak potential for the irreversible oxidation waves.

d *E*_pc_ refers to the cathodic peak potential for the irreversible reduction waves.

e *E*_HOMO_ levels were calculated from electrode potentials, i.e., *E*_HOMO_ = −[*E*_*pa*_ (vs. Fc^+^/Fc) + 4.80] eV or *E*_HOMO_ = −[*E*_1/2_^*ox*^ (vs. Fc^+^/Fc) + 4.80] eV. Eº(Fc^+^/Fc) = +0.46 V versus SCE in CH_2_Cl_2_ (0.1 M ^*n*^Bu_4_NPF_6_) [42].

f *E*_LUMO_ levels were calculated from electrode potentials, i.e., *E*_LUMO_ = −[*E*_*pc*_ (vs. Fc^+^/Fc) + 4.80] eV or *E*_LUMO_ = −[*E*_1/2_^*red*^ (vs. Fc^+^/Fc) + 4.80] eV. Eº(Fc^+^/Fc) = +0.46 V versus SCE in CH_2_Cl_2_ (0.1 M ^*n*^Bu_4_NPF_6_) [42].

### 2.4 Computational Studies

**FIGURE 5:**
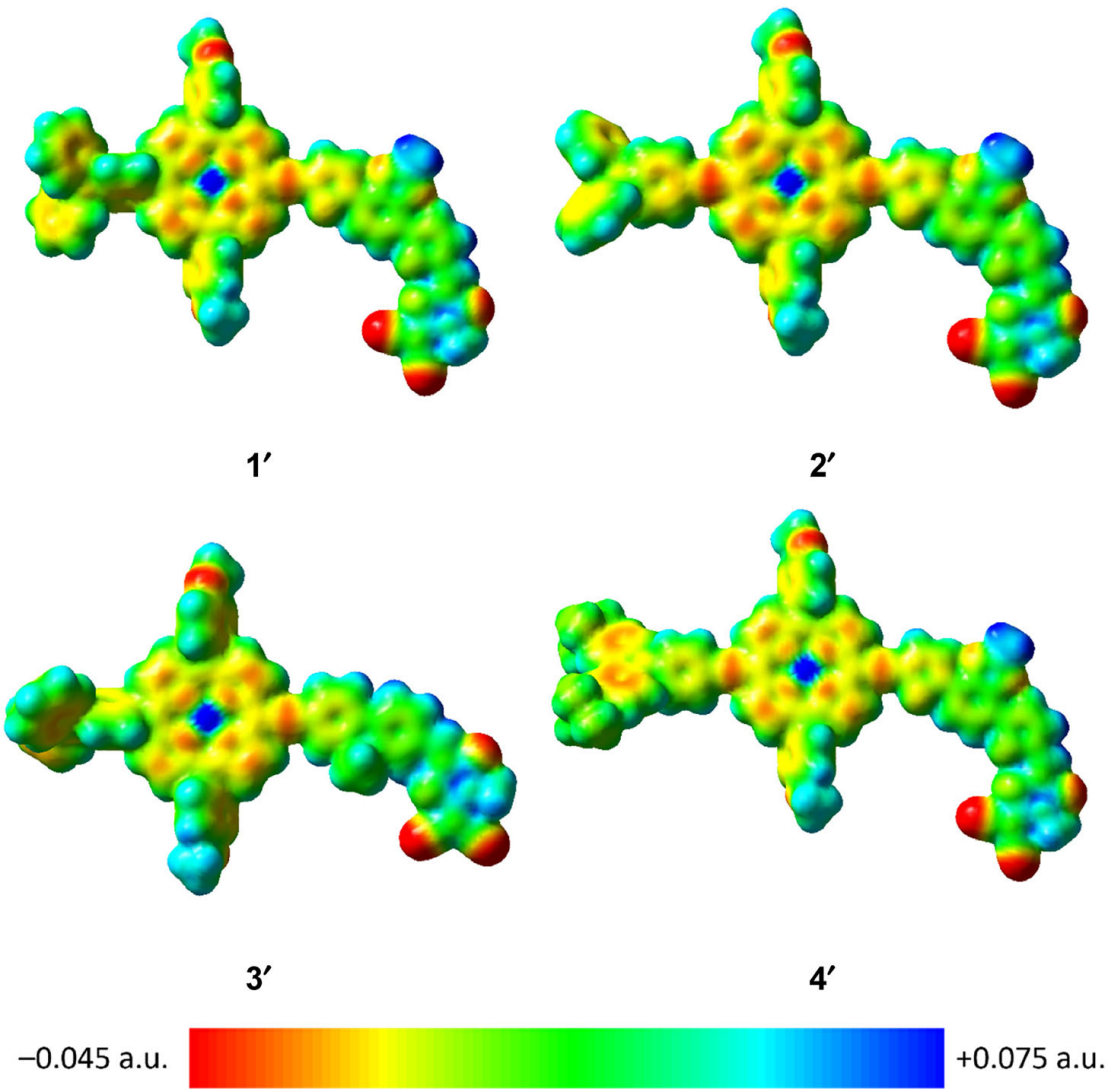
ESP surfaces (isovalue = 0.02) of **1** **′**−**4** **′**. The most negative potential is colored in red and the most positive potential is colored in blue.

To further investigate the photophysical and electronic properties of **1**−**4**, the density functional theory (DFT) and time‐dependent DFT (TDDFT) calculations have been performed on the model complexes of **1**−**4** (labeled as **1′**−**4′**), where the −^*n*^C_6_H_13_ and −^*n*^C_2_H_3_(C_2_H_5_)(C_4_H_9_) groups are replaced by −CH_3_ groups to reduce the computational cost. Figure S4 displays the optimized ground‐state (S_0_) geometries of **1′**−**4′**, and their corresponding Cartesian coordinates are given in Tables S4−S7. It is observed that, for **1′**−**4′**, the π‐bridges and acceptor moieties are almost coplanar. While **1′** and **3′** display similar dihedral angles of > 62° between the donor and porphyrin moieties, **2′** and **4′** show a much smaller dihedral angle of < 5°, suggesting the effective planarization of these complexes with the addition of −C≡C− moieties between the donor and porphyrin units. Table S3 shows the first ten singlet−singlet transitions of **1′**−**4′** computed by the TDDFT/SMD method. Selected molecular orbitals involved in the transitions are shown in Figures S5−S8, and simulated UV–vis spectra are shown in Figures S9−S12. The lower‐energy absorption bands of **1′**−**4′** at *ca.* 653−780 nm, corresponding to the S_1_ excitation associated with the HOMO → LUMO excitation. The HOMOs of **1′**−**4′** are the π‐orbitals mainly located on the donor and porphyrin moieties, while the LUMOs are the π*‐orbitals mainly located on the π‐bridge and acceptor moieties. Therefore, the low‐energy absorption bands are assigned as the ICT transitions from the donor and porphyrin to the π‐bridge and acceptor, supporting the spectral assignments of the UV–vis absorption spectra. Moreover, the electrostatic potential (ESP) surfaces of these complexes have also been computed and are shown in Figure 5. The negative regions of the ESP surfaces are found to be mainly localized on the porphyrin units and are extended to the π‐bridge as well as the electron‐acceptor units. According to the previous studies, negative ESP regions may serve as a charge trap to impede the mobility of the charge carriers [37].

### 2.5 Characterization of Resistive Memory Devices

**FIGURE 6:**
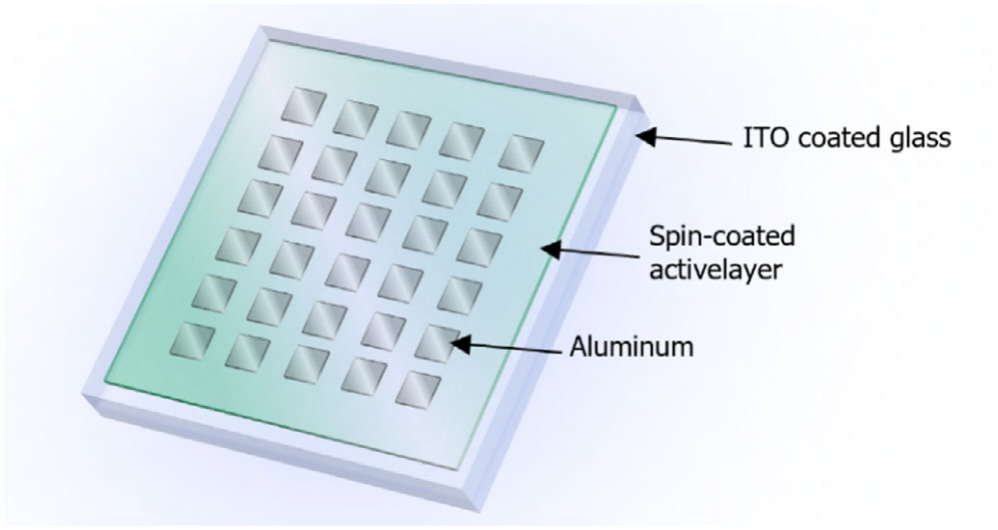
Schematic illustration of memory device structure.

**FIGURE 7:**
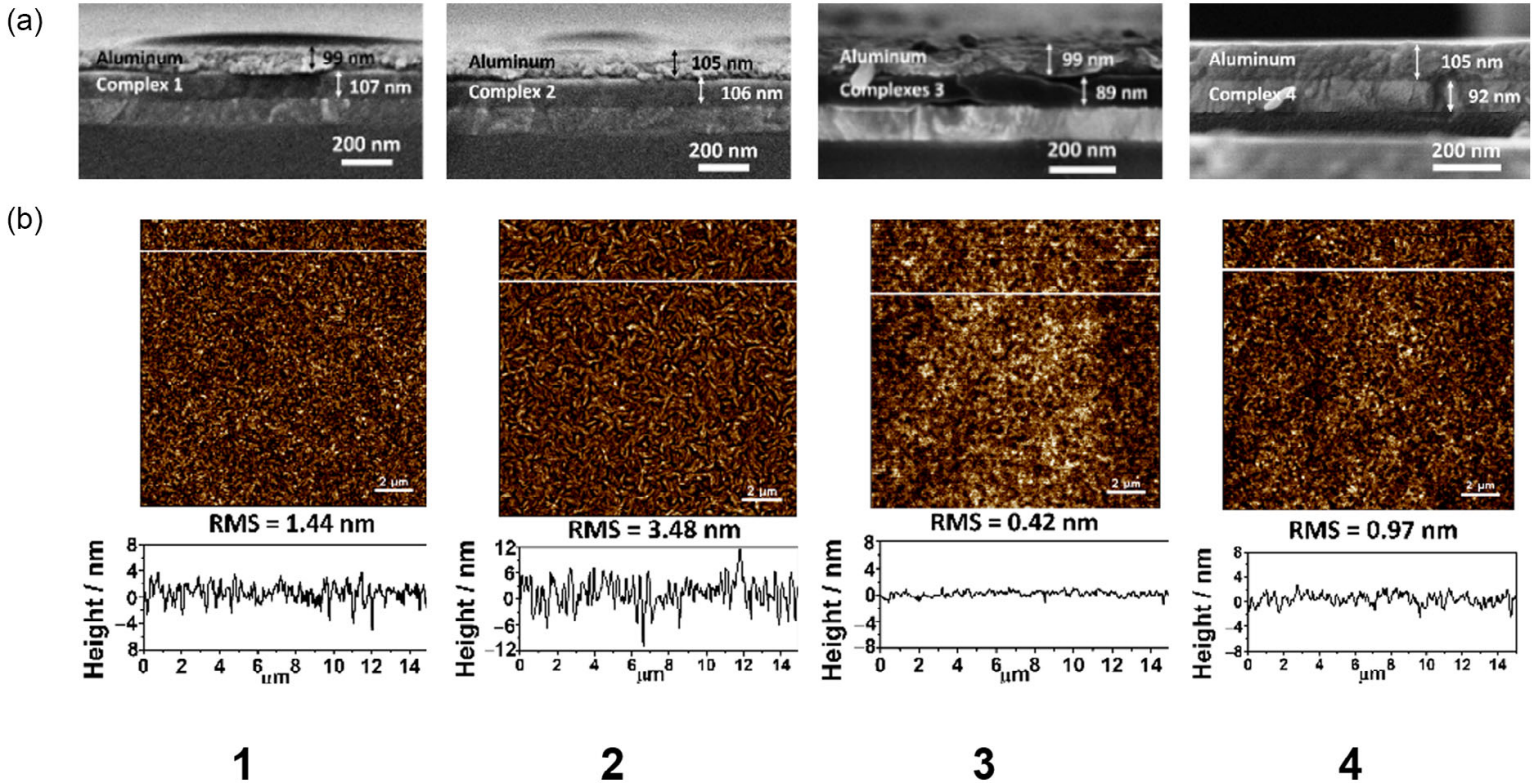
(a) SEM image of a cross‐section of the devices based on complexes **1**−**4** and (b) tapping‐mode (15 × 15 μm) AFM image (top) and the corresponding cross‐section profile of the AFM topographic image (bottom) of the spin‐coated film of **1**−**4**.

In order to explore the potential application of these complexes in resistive memory devices, complexes **1**−**4** have been fabricated with the sandwich device structure of Al/lithium fluoride (LiF)/**1**−**4**/indium‐tin‐oxide (ITO) (Figure 6). These complexes were dissolved in chloroform and spin‐coated on the ITO‐coated borosilicate glass, in which the ITO layer serves as the anode of the memory device. LiF was vacuum‐deposited onto the active layer as protecting layer to prevent filamentary conduction. Lastly, aluminum was thermal‐deposited as the cathode [44]. As shown in the scanning electron microscopic (SEM) images of the cross‐sections of the memory devices (Figure 7a), the thicknesses of the active layer of **1**−**4** and aluminum are found to be in the range of 89−107 nm and 99−105 nm, respectively. It is noteworthy that **1**−**4** exhibit good film formation properties, and homogeneous films of high optical transparency have been formed. In addition, the spin‐coated films of **1**−**4** possess a smooth film surface with a small root‐mean‐square roughness of ±0.42−±3.48 nm, as shown by atomic force microscopy (AFM) (Figure 7b).

**FIGURE 8:**
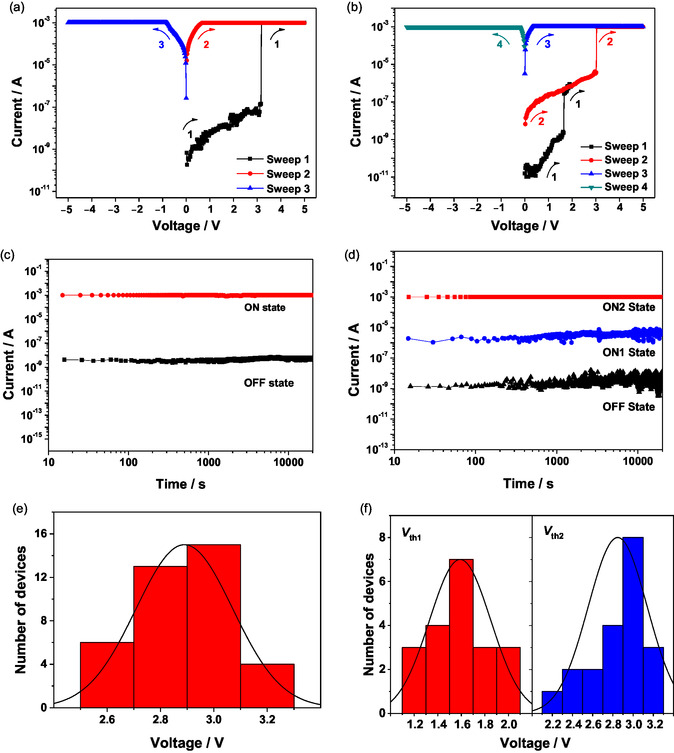
Representative current–voltage characteristics of an (a) ITO/LiF/**1**/Al device. (b) ITO/LiF/**3**/Al device. (c) Retention time of the memory devices fabricated with **1** in “OFF” and “ON1” states under constant stress (1.0 V). (d) Retention time of the memory devices fabricated with **3** in “OFF”, “ON1”, and “ON2” states under constant stress (1.0 V). Bar charts of the number of devices based on (e) **1** against threshold voltage of “ON” state among 38 devices and (f) **3** against threshold voltage of “ON” state among 20 devices.

**FIGURE 9:**
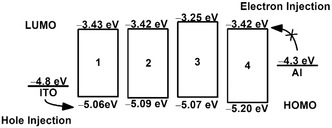
Schematic diagram of the charge injection process in **1** **−** **4** based resistive memory devices.

The current−voltage (*I*−*V*) characteristics of the memory devices based on **1**−**4** have been investigated and are shown in Figures 8 and S13, S14. Two electrically stable states are observed in devices based on **1** and **2** in the same range of voltage bias, indicating a binary memory behavior. The *V*_th_ of **1** and **2** are found to be at *ca.* 3.0 and *ca.* 2.2 V, respectively (Figures 8a and S13a). Meanwhile, the “OFF” and “ON” current ratios of 1:10^5^ and 1:10^6^ are observed for these memory devices based on **1** and **2**, respectively. Both devices show stable “ON” and “OFF” states under the constant voltage test with no significant degradation over 20,000 s (Figures 8c and S13b). Interestingly, with a change in the π‐bridge (**3**) or the donor (**4**), ternary memory behaviors can be achieved. For **3**, when the applied voltage was increased from 0 to 2 V, a sudden increase in current from 10^−9^ to 10^−6^ A was found at the first switching threshold voltage (*V*_th1_) of *ca.* 1.6 V, implying a transition from a high‐resistance “OFF” state to an intermediate‐resistance “ON1” state (Sweep 1) (Figure 8b). The intermediate‐resistance “ON1” state can be maintained during the subsequent sweep from 0 to 3 V (Sweep 2), while a second switching threshold voltage (*V*_th2_) was found at *ca.* 3.0 V, indicating a transition from the intermediate‐resistance “ON1” state to a low‐resistance “ON2” state. The device remained in the “ON2” state in the third sweep from 0 to 5 V (Sweep 3), and the stability of the “ON2” state has been demonstrated by applying an inverse sweep from 0 to −5 V (Sweep 4), in which the “ON2” state remained. The “ON2” state was found to be relaxed to the “OFF” state after switching off the power, demonstrating a volatile memory behavior. The reset process occurs by self‐relaxation upon switching off the voltage, as the charges would de‐trap from the porphyrin core and charge‐separated states would be deactivated, resulting in the reversion to the high‐resistance “OFF” state. The two discrete transitions from “OFF” to “ON1” and “ON2” can be regarded as two different “writing” processes of the memory devices. The current ratios of the three different resistive “OFF”, “ON1” and “ON2” states are found to be 1:10^4^:10^6^, suggestive of a well‐separated ternary memory behavior of the device. Durability tests on these devices were performed by applying a constant voltage of 1 V, with the three resistive states showing stable currents of over 20,000 s (Figure 8d). Furthermore, consistent memory performance with well‐separated narrow distributions of *V*_th1_ and *V*_th2_ based on complex **3** is observed (Figure 8f), suggesting a good reproducibility of these ternary memory devices. Moreover, the memory behavior of **4** has also been investigated, and the *I–* *V* characteristics are shown in Figure S14a. Upon applying a voltage from 0 to 5 V, the *V*_th1_ and *V*_th2_ are shown at *ca.* 1.6 and 2.2 V, respectively, and the current ratios of “OFF”, “ON1”, and “ON2” states are found to be 1:10^4^:10^5^. The constant stress test of **4** has also been performed. There was no significant degradation of the three different states for over 20,000 s under a constant voltage of 1 V (Figure S14b). Consistent memory performance has also been observed, with a narrow distribution of *V*_th1_ and *V*_th2_ among 20 devices, indicating the high reproducibility of the ternary memory performance (Figure S14c). Based on the electrochemical data determined from cyclic voltammetry, the energy levels of the HOMO and LUMO of **1**−**4** are found to be in the range of −5.06 to −5.20 eV and −3.25 to −3.43 eV, respectively (Figure 9). The energy barriers for the hole and electron injection, estimated by the energy difference between the ITO electrode (−4.8 eV) and the HOMO energy and between the Al electrode (−4.3 eV) and the LUMO energy, are 0.26−0.40 eV and 0.87−1.05 eV, respectively. The smaller energy barriers for hole injection when compared to electron injection suggested that hole injection is more energetically favorable from the ITO electrode to the active layer.

**FIGURE 10:**
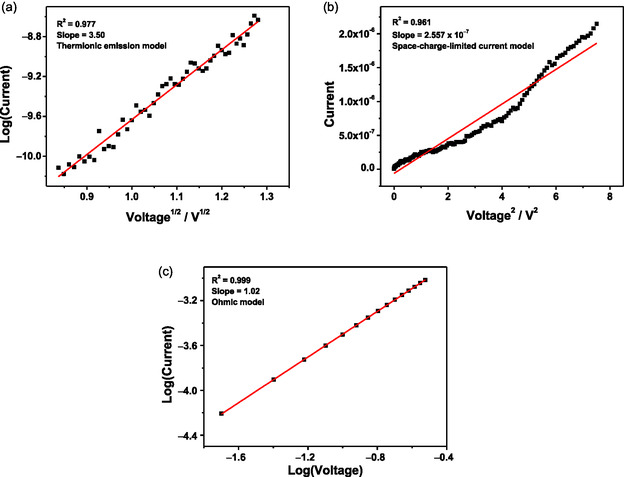
(a) Plots of log(*I*) versus *V* ^1/2^ obtained by fitting the *I*−*V* characteristics of the “OFF” state (from 0.7 to 1.64 V). (b) Plots of *I* versus *V* ^2^ obtained by fitting the *I*−*V* characteristics of the “ON1” state (from 0.02 to 2.74 V). (c) Plots of log(*I*) versus log(*V*) obtained by fitting the *I*−*V* characteristics of the “ON2” state (from 0.02 to 0.3 V) of the memory device fabricated with **3**.

To gain more understanding of the switching mechanism, the *I*−*V* curves of **1**−**4** at different resistive states have been fitted with different charge transport models (Figures 10 and S15−S17) [45]. For all the memory devices at the “OFF” states, the *I* *−V* curves can be appropriately fitted with the log(*I*) versus *V* ^1/2^ charge transport models, indicating the involvement of a thermionic emission process [38, 46, 47, 48, 49]. Meanwhile, the *I* *−V* curves of the “ON1” state of ternary memory devices based on **3** and **4** can be fitted to the space‐charge‐limited current conduction model [*I* vs. *V* ^2^] [38, 47, 49, 50]. For the “ON” state of **1** and **2**, as well as the “ON2” state of **3** and **4**, they can be fitted with the ohmic conduction model [log(*I*) vs. log(*V*)] [45, 48, 49, 50]. According to previously reported literature, the differences in charge‐transport models of these binary and ternary memory devices may be associated with different switching mechanisms in different resistive states [45, 51].

The intriguing memory behavior difference between **1**−**2** (binary) and **3**−**4** (ternary) may originate from their different chemical structures. Comparatively, **1** and **3** bear the same donor and acceptor moieties, while **1** has an electron‐withdrawing π‐bridge and **3** has an electron‐donating π‐bridge. A ternary memory behavior has been found in **3** but not in **1**. By reviewing the absorption data of **1** and **3**, the Q‐band of **3** (629 nm) is blue‐shifted when compared to **1** (650 nm), suggesting a weaker ICT character [41]. Thus, the use of an electron‐donating π‐bridge may weaken the electronic interaction between the donor and acceptor moieties [41]. Therefore, a relatively long‐lived charge‐separated state could be generated [52, 53]. As a result, a field‐induced charge transfer state from the triphenylamine to the 9,9‐dihexylfluorene π‐bridge and acceptor is created, which is responsible for the switching from the “OFF” state to the “ON1” state of **3**. In addition, the similar *V*_th_ of **1** (*ca.* 3.0 V) and *V*_th2_ of **3** (*ca.* 3.0 V) may suggest that both states involve the same switching mechanism, such that the *I–* *V* curves of “ON” state of **1** and “ON2” state of **3** can be fitted with the same ohmic conduction model. In view of the structural similarity as well as the negative ESP surface, the “ON” state of **1** and “ON2” state of **3** are proposed to be derived from the charge‐trapping mechanism originating from the porphyrin core. Once the charge trap sites are filled with the injected charges, the newly injected charges can bypass the barriers of charge traps, reducing the resistance, leading to a higher current flow state [5]. Similar to **3**, it is proposed that the incorporation of a weaker electron‐donating *t*‐butylcarbazole unit in **4** would induce a long‐lived charge transfer process from the donor to the acceptor, which is responsible for the switching from the “OFF” state to “ON1” state of **4** [53]. Similar to the case of **1** and **3**, the *V*_th_ of **2** (*ca.* 2.2 V) and *V*_th2_ of **4** (*ca.* 2.2 V) are found to be similar. Due to their similar structure, ESP surface studies and the fitting of *I–* *V* curves with charge transport models, these complexes are proposed to have similar switching mechanisms, and therefore the “ON” state of **2** and “ON2” state of **4** are assigned as originating from the charge‐trapping states contributed by the porphyrin core. These results show that the incorporation of an electron‐donating π‐bridge or a weak donor can induce an independent charge transfer process, turning on an extra resistance state “ON1” without affecting the original charge‐trapping “ON” and “ON2” states. Similar ternary memory behavior was also recently reported in porphyrins with weak electron‐donating and withdrawing couples substituted at the *β*‐positions, further supporting the potential of weak D−Por−A systems to realize multilevel memory behavior [37]. Compared to the reported multilevel memory devices, the presence of strong electron‐donating and withdrawing groups is important for achieving the third or fourth resistive state [20, 38]. The present study has systematically studied the effect of donor and π‐bridge from binary to ternary memory behavior by judicious molecular design of metalloporphyrin complexes. More importantly, it demonstrates a new direction for the realization of ternary memory performance.

## 3 Conclusion

A series of zinc(II) porphyrin‐based D−Por−π−A and D−π−Por−π−A complexes have been designed, synthesized, characterized, and employed in solution‐processable resistive memory devices. This study demonstrates the relationship between memory behavior and molecular structure, in which the utilization of electron‐donating π‐bridge or weak electron‐donating moiety is able to turn on an extra resistive state. The ternary memory performances of the devices have been demonstrated with distinct *V*_th_, high current ratio, and good stability of conductive states. Through the correlation of molecular structure, photophysical, electrochemical, and computational studies, the two distinct resistive states have been assigned as attributed to the charge transfer process from the donor to the acceptor moieties and the charge‐trapping of the zinc(II) porphyrin core, respectively. The work provides inspirational insight into the future design solution‐processable multilevel resistive memory materials.

## Supporting Information

Additional supporting information can be found online in the Supporting Information section.

## Author Contributions

**Vivian Wing‐Wah Yam:** conceptualization, methodology, formal analysis, supervision, project administration, funding acquisition, writing – review & editing, resources, investigation, validation. **Ka Wai Kwong:** writing – original draft, investigation, methodology, formal analysis, validation, data curation. **Hing Chan:** investigation, methodology, validation, formal analysis, data curation. **Ming‐Yi Leung:** methodology, formal analysis, validation, investigation, data curation, writing – original draft. **Eugene Yau‐Hin Hong:** methodology, validation, investigation, formal analysis, data curation. **Shiu‐Lun Lai:** methodology, investigation.

## Funding

This study was supported by Innovation and Technology Commission (ITC) (Hong Kong Quantum AI Lab Ltd under the AIR@InnoHK cluster), General Research Fund (GRF) of the Research Grants Council of the Hong Kong Special Administrative Region, People's Republic of China (HKU 17312422).

## Conflicts of Interest

The authors declare no conflicts of interest.

## Supporting information

Supplementary Material

## Data Availability

The data that supports the findings of this study are available in the supplementary material of this article.
