# Supplementary material for: Molecular Design Toward High‐Performance Solution‐Processable Push–Pull Zinc(II) Porphyrin‐Based Resistive Memory Devices: From Binary to Ternary Memory Behavior
Source: Small Sci. 2026 May 15;6(5):e70285. doi: 10.1002/smsc.70285 (PMC13176949; doi:10.1002/smsc.70285)
Supplement: Supplementary file 1 — Supplementary Material [file SMSC-6-e70285-s001.pdf]

Molecular Design Toward High-Performance Solution-Processable Push-Pull Zinc(II) Porphyrin-Based Resistive Memory Devices: From Binary to Ternary Memory Behavior

Ka Wai Kwong,[ab] Hing Chan,[a] Ming-Yi Leung,[ab] Eugene Yau-Hin Hong,[a] Shiu-Lun Lai,[a] and Vivian Wing-Wah Yam*[ab]

[a]Department of Chemistry, The University of Hong Kong, Pokfulam Road, Hong Kong, P. R. China. Fax: +(852) 2857-1586; Tel: +(852) 2859-2153

E-mail: wwyam@hku.hk

[b]Hong Kong Quantum AI Lab Limited, 17 Science Park West Avenue, Pak Shek Kok, Hong Kong, P. R. China

Supporting Information

Table of Content

		Page
**Physical Measurements and Instrumentation**		S5
**Fabrication and Measurements of the Memory Devices**		S6
**Synthesis and Characterization**		
**Scheme S1.**	Synthetic route of complex **1**.	S7
**Scheme S2.**	Synthetic route of complex **2**.	S8
**Scheme S3.**	Synthetic route of complex **3**.	S8
**Scheme S4.**	Synthetic route of complex **4**.	S9
**Photophysical Properties**		
**Table S1.**	Electronic absorption properties of push–pull metalloporphyin **1**–**4** in toluene and solid-state thin film at 298 K	S12
**Table S2.**	Emission data of push–pull zinc(II) porphyrin **1**–**4**	S13
**Figure S1.**	Electronic absorption spectra of **1**–**4** in toluene solution and in neat film.	S14
**Figure S2.**	Solvent-dependent UV-visible absorption spectra of **2**.	S15
**Figure S3.**	Normalized solvent-dependent emission spectra of **2**.	S15
**Computational Studies**		
**Figure S4.**	Optimized ground-state geometries with selected structural parameters of **1′**–**4′**.	S17
**Table S3.**	The first ten singlet excitations (S_n) of **1′**–**4′** computed by TDDFT/DCM using toluene as the solvent	S18
**Figure S5.**	Spatial plots (isovalue = 0.03) of selected molecular orbitals of **1′** at the optimized ground-state geometry.	S21
**Figure S6.**	Spatial plots (isovalue = 0.03) of selected molecular orbitals of **2′** at the optimized ground-state geometry.	S21
**Figure S7.**	Spatial plots (isovalue = 0.03) of selected molecular orbitals of **3′** at the optimized ground-state geometry.	S22

**Figure S8.**	Spatial plots (isovalue = 0.03) of selected molecular orbitals of **4′** at the optimized ground-state geometry.	S22
**Figure S9.**	Simulated UV–vis spectrum of **1′** computed by TDDFT/CPCM using dichloromethane as the solvent.	S23
**Figure S10.**	Simulated UV–vis spectrum of **2′** computed by TDDFT/CPCM using dichloromethane as the solvent.	S23
**Figure S11.**	Simulated UV–vis spectrum of **3′** computed by TDDFT/CPCM using dichloromethane as the solvent.	S24
**Figure S12.**	Simulated UV–vis spectrum of **4′** computed by TDDFT/CPCM using dichloromethane as the solvent.	S24
**Table S4**	Cartesian coordinates of the optimized ground–state geometry of **1′**.	S25
**Table S5**	Cartesian coordinates of the optimized ground–state geometry of **2′**.	S26
**Table S6**	Cartesian coordinates of the optimized ground–state geometry of **3′**.	S27
**Table S7**	Cartesian coordinates of the optimized ground–state geometry of **4′**.	S28

Memory Properties

**Figure S13.**	(a) Representative current–voltage characteristics of an ITO/LiF/**2**/Al device. (b) Retention time of the memory devices fabricated with **2** in "OFF"and "ON" states under constant stress (1.0 V). (c) Bar chart of the number of devices against threshold voltage of "ON" state among 23 devices.	S31
**Figure S14.**	(a) Representative current–voltage characteristics of an ITO/LiF/**4**/Al device. (b) Retention time of the memory devices fabricated with **4** in "OFF", "ON1" and"ON2" states under constant stress (1.0 V). (c) Bar charts of the number of devices against threshold voltage of "ON" state among 20 devices.	S32

Figure S15. (a) Plot of log(*I*) *vs*. $V^{1/2}$ obtained by fitting the *I*–*V* characteristics of the "OFF" state (from 0.18 to 3.12 V) and (b) plot of log(*I*) *vs*. log(*V*) obtained by fitting the *I*–*V* characteristics of the "ON" state (from 0.02 to 0.7 V) of the memory device fabricated with **1**. S33

Figure S16. (a) Plot of log(*I*) *vs*. $V^{1/2}$ obtained by fitting the *I*–*V* characteristics of the "OFF" state (from 0.68 to 2.26 V) and (b) plot of log(*I*) *vs*. log(*V*) obtained by fitting the *I*–*V* characteristics of the "ON" state (from 0.02 to 0.14 V) of the memory device fabricated with **2**. S33

Figure S17. (a) Plot of log(*I*) *vs*. $V^{1/2}$ obtained by fitting the *I*–*V* characteristics of the "OFF" state (from 0.12 to 1.44 V). (b) Plot of *I* *vs*. V^2 obtained by fitting the *I*–*V* characteristics of the "ON1" state (from 0.64 to 2.44 V). (c) Plot of log(*I*) *vs*. log(*V*) obtained by fitting the *I*–*V* characteristics of the "ON2" state (from 0.02 to 0.72 V) of the memory device fabricated with 4. S34

References S35

Physical Measurement and Instrumentation

^1H NMR spectra were recorded on a Bruker AV 500 Fourier transform NMR spectrometer with chemical shifts recorded relative to tetramethylsilane (Me_4Si). High-resolution electrospray ionization (ESI) mass spectroscopy was recorded on a Bruker MaXis II Ultrahigh-Resolution Time-of-Flight (QTOF) Mass Spectrometer. Elemental analyses were performed on the Carlo Erba 1106 elemental analyzer at the Institute of Chemistry, Chinese Academy of Sciences (Beijing, China). All solution samples for photophysical studies were freshly prepared under a high vacuum in a round-bottomed flask equipped with a side-arm 1-cm fluorescence cuvette and sealed from the atmosphere by a Rotaflo HP6/6 quick-release Teflon stopper. Toluene solution of **1**–**4** was prepared to investigate their absorption and photoluminescence (PL) characteristics in the solution state. Solutions were degassed by using a high vacuum line in a two-compartment cell with four freeze-pump-thaw cycles. The UV-vis absorption spectra were recorded on a Varian Cary 50 spectrophotometer equipped with a Xenon flash lamp. Steady-state emission spectra were recorded using a Edinburgh Instruments FS5 spectrofluorometer. Relative luminescence quantum yields in solution were measured by the optical dilute method reported by Demas and Crosby[1] with a degassed aqueous solution of $[Ru(bpy)_3]Cl_2$ (Φ = 0.042, excitation wavelength at 436 nm) as the reference.[2] Solid-state photophysical measurements were carried out with the solid sample loaded in a quartz tube inside a quartz-walled Dewar flask. Liquid nitrogen was placed into the optical Dewar flask for low temperature (77 K) photophysical measurements. The excited-state lifetimes of solution samples were measured with an Edinburgh Instruments LP980 spectrometer, while that of solid and glass samples were measured using a conventional laser system. The excitation source used was the 355-nm output (third harmonic, 8 ns) of a Spectra-Physics Quanta-Ray Q-switched GCR-150 pulsed Nd:YAG laser (10 Hz). Cyclic voltammetric measurements were performed by using a CH Instruments, Inc. model CHI 620A electrochemical analyzer. The electrolytic cell used was a conventional two compartment cell. Electrochemical measurements were performed in acetonitrile solutions with 0.1 M $^nBu_4NPF_6$ as supporting electrolyte at room temperature. The reference electrode was a Ag/$AgNO_3$ (0.1 M in acetonitrile) electrode, and the working electrode was a glassy carbon electrode (CH Instruments, Inc.) with a platinum wire as the counter electrode. The working electrode surface was first polished with 1 μm alumina slurry (Linde),

followed by 0.3 μm alumina slurry, on a microcloth (Buehler Co.). The ferrocenium/ferrocene couple (Fc^+/Fc) was used as the internal reference. All solutions for electrochemical studies were deaerated with prepurified argon gas pripor toe measurements.

AFM Studies

The AFM images were obtained using a Bruker NanoWizard ULTRA Speed 2. The samples were prepared by spin-coating the sample solution onto a quartz plate.

Electron Microscopy

SEM experiments were performed on a Hitachi S-4800 FEG Scanning Electron Microscope at the Electron Microscope Unit of The University of Hong Kong.

Fabrication and Measurements of the Memory Devices

The transparent anode indium-tin-oxide (ITO)-coated borosilicate glass substrate (2 cm × 2 cm) was ultrasonicated successively with deionized water, acetone, isopropanol and absolute ethanol for 15 min each and then dried in an oven at 120 °C for an hour. Chloroform solutions of compounds **1**–**4** (*ca.* 10 mg mL^{-1}) were spin-coated onto the ITO substrate followed by thermal annealing at 75 °C for 15 min. The deposition of a thin-layer (*ca.* 5 nm) of protecting layer, lithium fluoride (LiF), was achieved by thermal evaporation under a pressure of around 5×10^{-6} mbar. Each organic surfaces were deposited with an aluminum top electrode (*ca.* 100 nm) by thermal evaporation through a shadow mask under a pressure of around 5×10^{-6} mbar. 400 devices were fabricated on each ITO glass platform and the active area of each cell was about 0.25 mm^2. Current−voltage (I−V) characteristics of the memory devices were measured with a programmable Keithley model 4200SCS power source in a probe station. All electrical measurements of the device were taken under ambient conditions.

Synthesis and Characterization

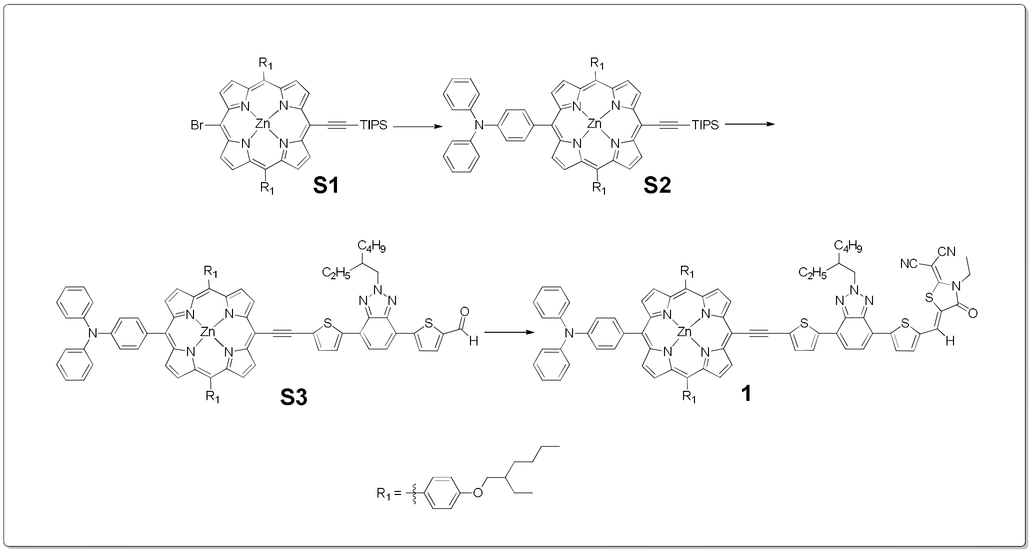

Scheme S1. Synthetic route of complex **1**.

Complex **S1** was prepared by a modified reported method.[3] **S2** was synthesized by Suzuki coupling of **S1**, $Pd(PPh_3)_4$, 4-(4,4,5,5-tetramethyl-1,3,2-dioxaborolan-2-yl)triphenylamine and K_2CO_3 in the presence of THF and water mixture. π-bridges were prepared by the previously reported method.[4–6] **S3** were synthesized by deprotection of **S2** in 1.0 M tetra-*n*-butylammonium fluoride (TBAF) in tetrahydrofuran (THF) and added to the degassed THF and diisopropylamine solution containing $Pd(PPh_3)_4$, SPhos and the corresponding π-bridges and stirred at reflux for 24 h. The final complex **1** were synthesized by Knoevenagel condensation. **S3** and 2-(1,1-dicyanomethylene)-3-ethylrhodanine (CNR), were added into chloroform solution and heated to 50 °C, followed by a few drops of piperidine. The reactions were heated to reflux and monitored by TLC. Once the reaction is completed, the reaction mixture was then extracted with chloroform and washed with deionized water. The organic layer was dried over anhydrous magnesium sulfate, filtered and evaporated to dryness under reduced pressure. The crude product was then purified by flash column chromatography on silica gel using dichloromethane as eluent. Subsequent recrystallization by the layering of methanol or hexane onto the concentrated toluene solution of the compound gave the resulting complex.

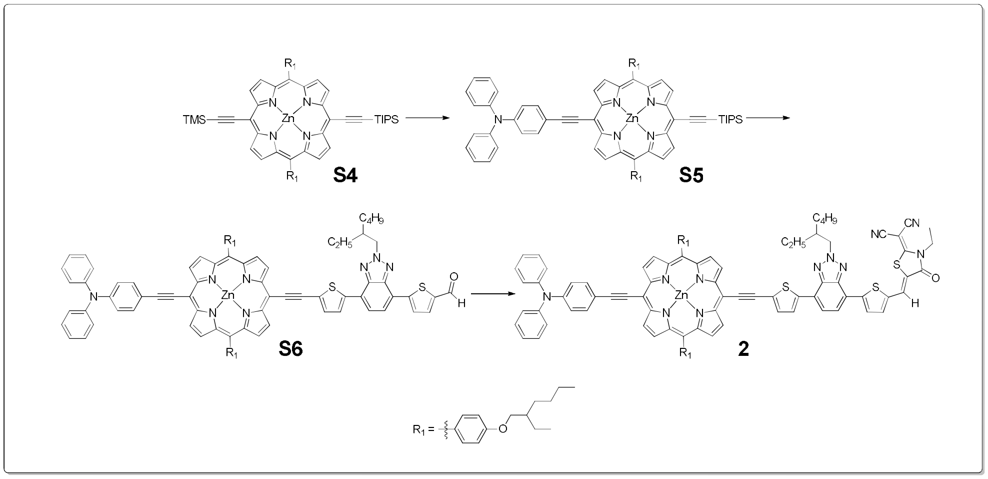

Scheme S2. Synthetic route of complex **2**.

S4 was prepared by Sonogashira coupling of **S1** with trimethylsilylacetylene, followed by deprotection with K_2CO_3 in THF solution. The deprotected **S4** was added to the degassed THF and diisopropylamine solution containing $Pd(PPh_3)_4$, SPhos and 4-bromotriphenylamine and stirred at reflux for 24 h to give **S5**. **S6** were synthesized by deprotection of **S5** with TBAD in THF solution and added to the degassed THF and diisopropylamine solution containing $Pd(PPh_3)_4$, SPhos and corresponding π-bridges and stirred at reflux for 24 h. Complex **2** was prepared by Knoevenagel condensation as described above.

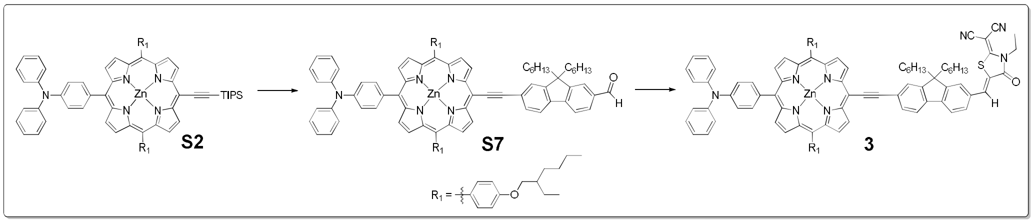

Scheme S3. Synthetic route of complex **3**.

The deprotected **S2** was added to the degassed THF and diisopropylamine solution containing $Pd(PPh_3)_4$, SPhos and corresponding π-bridges and stirred at reflux for 24 h to give **S7**. Complex **3** was prepared by Knoevenagel condensation as described above.

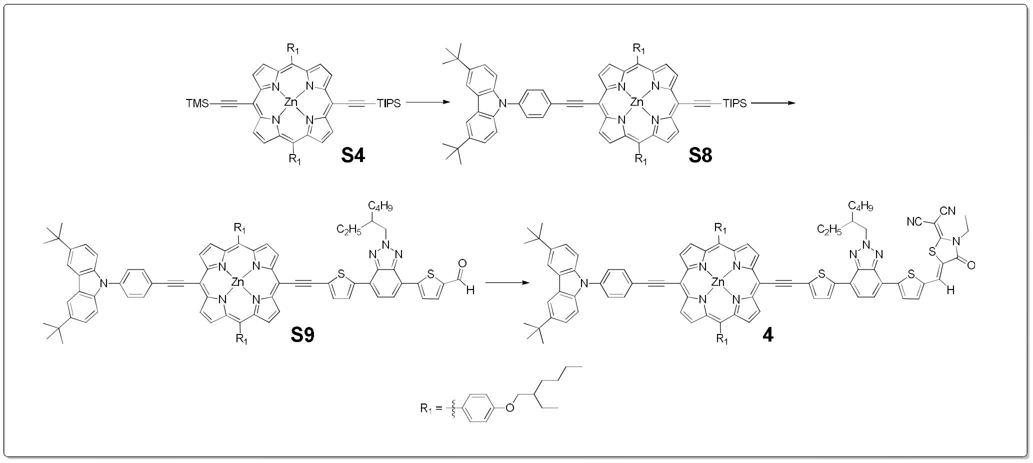

Scheme S4. Synthetic route of complex **4**.

S8 was prepared by deprotected **S4** and added to the degassed THF and diisopropylamine solution containing $Pd(PPh_3)_4$, SPhos and 3,6-di(*t*-butyl)-9-(4-bromophenyl)carbazole and stirred at reflux for 24 h. **S9** was synthesized by the deprotection of **S8**, followed by Sonogashira coupling with $Pd(PPh_3)_4$, SPhos and corresponding π-bridges in degassed THF and diisopropylamine solution and stirred at reflux for 24 h. Complex **4** was prepared by Knoevenagel condensation as described above.

1: Yield: 67 mg, 51 %. ^{1}H NMR (500 MHz, $CDCl_3$, 318 K, relative to Me_4Si, δ/ppm): δ 0.96–1.02 (m, 6H, $-CH_3-$), 1.08–1.13 (m, 9H, $-CH_3$), 1.18 (t, J = 7.5 Hz, 6H, $-CH_3$), 1.29 (s, 1H, $-CH_2-$), 1.40–1.46 (m, 3H, $-CH_2-$), 1.53–1.84 (m, 19H, $-CH_2-$), 2.00–2.08 (m, 3H, $-CH-$), 2.29–2.37 (m, 1H, $-CH_2-$), 3.29 (s, 2H, $-NCH_2-$), 4.26 (d, J = 5.5 Hz, 4H, $-OCH_2-$), 4.79 (d, J = 7.5 Hz, 2H, $-NCH_2-$), 5.74 (s, 1H, thienyl), 6.07 (s, 1H, thienyl), 6.71 (s, 1H, thienyl), 6.86 (s, 1H, thienyl), 7.16 (t, J = 7.5 Hz, 2H, TPA), 7.37–7.45 (m, 16H, TPA, phenyl and benzotriazolyl), 7.92 (d, J = 7.5 Hz, 2H, TPA), 8.0 (s, 1H, alkenyl), 8.16 (d, J = 8.0 Hz, 4H, phenyl), 8.82–8.87 (m, 4H, β-porphyrin), 8.92 (d, J = 4.5 Hz, 2H, β-porphyrin), 9.36 (s, 2H, β-porphyrin). HRMS (positive ESI) calcd for $C_{99}H_{93}N_{11}O_3S_3Zn$: m/z = 1643.5910 $[M]^+$; found m/z = 1643.5870 $[M]^+$. Elemental analyzes: Found (%): C 71.49, H 5.62, N 9.24. Calcd for $C_{99}H_{93}N_{11}O_3S_3Zn$: C 72.22, H 5.69, N 9.36.

2: Yield: 37 mg, 38 %. ^{1}H NMR (500 MHz, $CDCl_3$, 318 K, relative to Me_4Si, *δ*/ppm): *δ* 0.99–1.04 (m, 6H, $-CH_3$), 1.17 (t, *J* = 7.5 Hz, 9H, $-CH_3$), 1.26 (t, *J* = 7.5 Hz, 6H, $-CH_3$), 1.31–1.46 (m, 8H, $-CH_2-$), 1.58–1.92 (m, 16H, $-CH_2$), 2.04–2.13 (m, 3H, –CH–), 3.29 (s, 2H, $-NCH_2-$), 4.26–4.37 (m, 6H, $-NCH_2-$ and $-OCH_2-$), 4.97 (s, 2H, thienyl), 6.46 (s, 1H, thienyl), 6.76 (s, 1H, thienyl), 7.15–7.19 (m, 4H, TPA and benzotriazolyl), 7.26 (d, *J* = 8.0 Hz, 6H, phenyl and TPA), 7.36–7.39 (m, 8H, TPA), 7.63 (d, *J* = 8.0 Hz, 3H, alkenyl and TPA), 7.92 (d, *J* = 8.0 Hz, 4H, phenyl), 8.27 (s, 2H, *β*-porphyrin), 8.34 (s, 2H, *β*-porphyrin), 8.50 (s, 2H, *β*-porphyrin), 9.01 (s, 2H, *β*-porphyrin). HRMS (positive ESI) calcd for $C_{101}H_{93}N_{11}O_3S_3Zn$: *m*/*z* = 1667.5911 $[M]^+$; found *m*/*z* = 1667.5862 $[M]^+$. Elemental analyzes: Found (%): C 71.92, H 5.55, N 9.16. Calcd for $C_{101}H_{93}N_{11}O_3S_3Zn$: C 72.62, H 5.61, N 9.22.

3: Yield = 58 mg, 64 %. ^{1}H NMR (500 MHz, $CDCl_3$, 318 K, relative to Me_4Si, *δ*/ppm): *δ* 0.78 (t, *J* = 8.0 Hz, 6H, $-CH_3$), 0.83–0.89 (m, 9H, $-CH_3$), 1.01 (t, *J* = 8.0 Hz, 6H, $-CH_3$), 1.13–1.22 (m, 15H, $-CH_2-$), 1.29–1.75 (m, 21H, $-CH_2-$) 1.92–1.99 (m, 2H, $-CH_2-$), 4.17 (d, *J* = 7.0 Hz, 4H, $-OCH_2-$), 4.26 (d, *J* = 8.5 Hz, 2H, $-NH_2-$), 7.12–7.16 (m, 2H, TPA), 7.32 (d, *J* = 10.0 Hz, 4H, phenyl), 7.41 (d, *J* = 10.5 Hz, 8H, TPA), 7.44 (d, *J* = 10.5 Hz, 2H, TPA), 7.53–7.55 (m, 2H, TPA), 7.94 (t, *J* = 10.0 Hz, 2H, fluorenyl), 8.0 (s, 1H, fluorenyl), 8.01 (s, 1H, fluorenyl), 8.04 (d, *J* = 10.0 Hz, 2H, fluorenyl), 8.07 (d, *J* = 10.0 Hz, 1H, alkenyl), 8.12 (d, *J* = 10.0 Hz, 4H, phenyl), 8.95 (d, *J* = 5.5 Hz, 2H, *β*-porphyrin), 9.01 (d, *J* = 5.5 Hz, 2H, *β*-porphyrin), 9.08 (d, *J* = 5.5 Hz, 2H, *β*-porphyrin), 9.87 (d, *J* = 5.5 Hz, 2H, *β*-porphyrin). HRMS (positive ESI) calcd for $C_{102}H_{102}N_8O_3SZn$: *m*/*z* = 1553.6743 $[M]^+$; found *m*/*z* = 1553.6738 $[M]^+$. Elemental analyzes: Found (%): C 77.13, H 6.25, N 6.84. Calcd for $C_{102}H_{102}N_8O_3SZn$: C 77.27, H 6.48, N 7.07.

4: Yield = 27 mg, 34 %. ^{1}H NMR (500 MHz, $CDCl_3$, 318 K, relative to Me_4Si, *δ*/ppm): *δ* 0.99–1.23 (m, 6H, $-CH_3$), 1.11–1.14 (m, 6H, $-CH_3$), 1.23 (t, *J* = 6.8 Hz, 6H, $-CH_3$), 1.50 (s, 18H, ^{t}Bu), 1.54–1.89 (m, 26H, $-CH_2-$), 2.05–2.16 (m, 4H, $-CH_2-$), 3.87 (s, 2H, $-NCH_2-$), 4.30 (d, *J* = 5.3, 4H, $-OCH_2-$), 4.47 (s, 2H, $-NCH_2-$), 4,96 (s, 1H, thienyl), 5.63 (s, 1H, thienyl), 6.68 (s, 1H, thienyl), 6.89 (s, 1H, thienyl), 7.42 (d, *J* = 7.7 Hz, 5H, phenyl), 7.49–7.54 (m, 5H, carbazolyl, phenyl and benzotriazolyl), 7.72 (d, *J* = 6.9 Hz, 3H, carbazolyl and alkenyl), 7.97 (d, *J* = 7.7 Hz, 6H, phenyl), 8.08 (d, *J* =

6.9 Hz, 2H, carbazolyl), 8.51 (s, 2H, *β*-porphyrin), 8.61 (s, 2H, *β*-porphyrin), 8.70 (s, 2H, *β*-porphyrin), 9.22 (s, 2H, *β*-porphyrin). HRMS (positive ESI) calcd for $C_{109}H_{107}N_{11}O_3S_3Zn$: *m*/*z* = 592.5665 $[M]^{3+}$; found *m*/*z* = 592.5630 $[M]^{3+}$. Elemental analyzes: Found (%): C 73.25, H 6.27, N 8.45. Calcd for $C_{109}H_{107}N_{11}O_3S_3Zn$: C 73.52, H 6.06, N 8.65.

Photophysical Data

Table S1. Electronic absorption properties of **1**–**4** in toluene and solid-state thin film at 298 K

Complexes	Medium	Absorption λ_{max} / nm ($\varepsilon_{max} \times 10^{-4}$ / $dm^3\ mol^{-1}\ cm^{-1}$)
**1**	Toluene	309 (4.47), 453 (18.3), 496 sh (6.10), 540 (6.03), 650 (6.95)
	Thin film	310, 481, 563 sh, 676
**2**	Toluene	308 (4.77), 470 (21.7), 538 (6.25), 681 (10.1)
	Thin film	312, 493, 572 sh, 721
**3**	Toluene	309 (4.37), 451, (25.4), 487 sh (6.48), 573 (1.81), 629 (5.28)
	Thin film	307, 456, 577, 646
**4**	Toluene	330 (4.11), 461 (23.3), 505 (6.70), 539 (6.17), 675 (9.84)
	Thin film	334, 480, 571 sh, 664 sh, 705

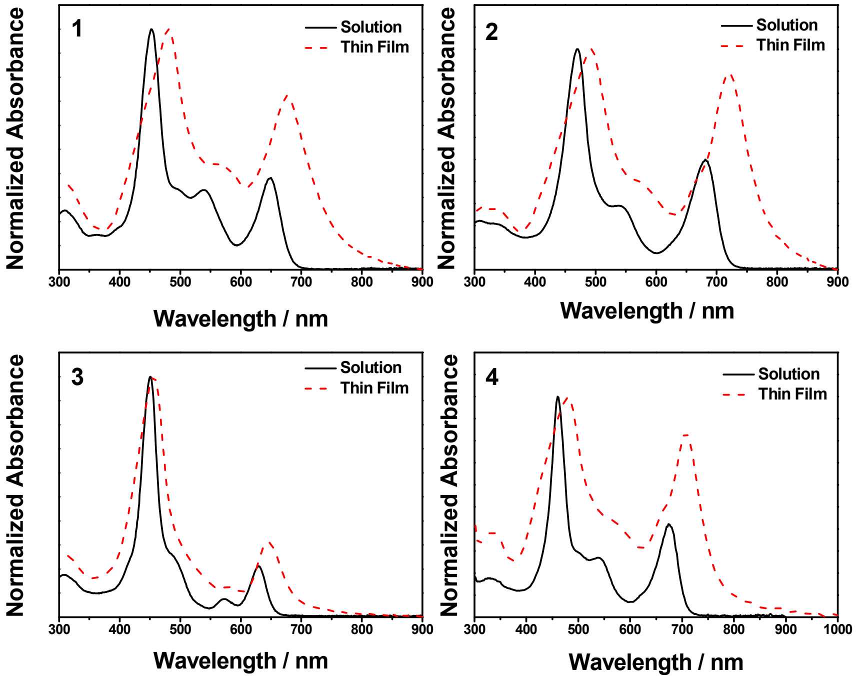

Figure S1. Electronic absorption spectra of **1**–**4** in toluene solution and neat film.

Table S2. Emission data of push–pull zinc(II) porphyrin **1–4**

Complexes	Medium (T/ K)	Emission λ_{max} / nm (τ_0 / μs)	Φ_{PL} [a]
**1**	Toluene (298)	683, 752 sh (>0.1μs)	0.12
	Solid (298)	— [d]	
	Solid (77)	— [d]	
	Glass (77) [b, c]	719, 767	
**2**	Toluene (298)	708, 779 sh (>0.1μs)	0.07
	Solid (298)	— [d]	
	Solid (77)	— [d]	
	Glass (77) [b, c]	753, 807	
**3**	Toluene (298)	652, 712 sh (>0.1μs)	0.10
	Solid (298)	— [d]	
	Solid (77)	— [d]	
	Glass (77) [b, c]	678, 732	
**4**	Toluene (298)	698, 761 (>0.1μs)	0.08
	Solid (298)	— [d]	
	Solid (77)	— [d]	
	Glass (77) [b, c]	747, 802	

[a] Relative luminescence photoluminescence quantum yield, measured at room temperature using $[Ru(bpy)_3]Cl_2$ in aqueous state as the reference.

[b] Measured in 2-methyltetrahydrofuran glass.

[c] Vibronic-structured emission band.

[d] Non-emissive

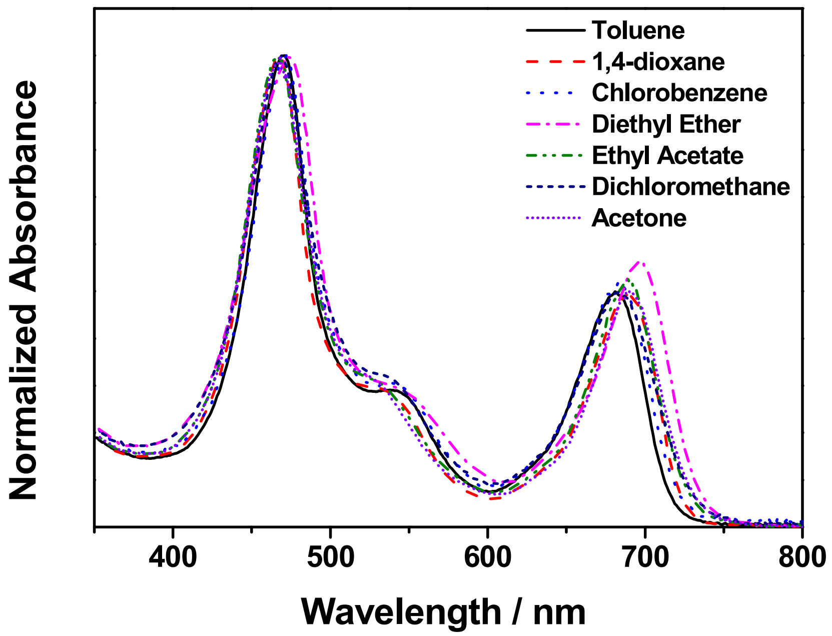

Figure S2. Normalized solvent-dependent UV-visible absorption spectra of **2**.

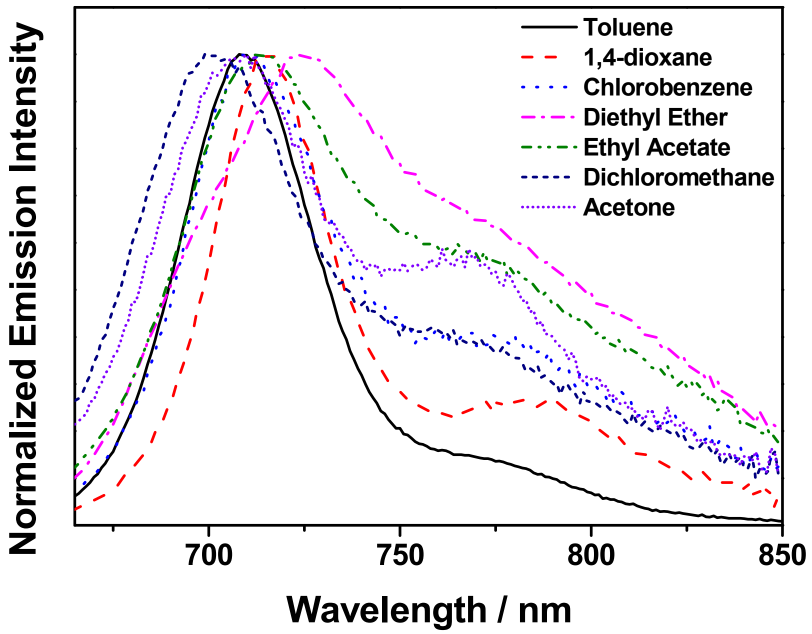

Figure S3. Normalized solvent-dependent emission spectra of **2**.

Computational Details

All calculations were carried out with the Gaussian 16 program suite.[7] The ground state (S_0) geometries of model compounds **1**–**4**, in which all the ethylhexyl and hexyl groups were replaced by methyl groups (labelled as **1'**–**4'**), were fully optimized at density functional theory (DFT) with the M06 functional[8] in conjunction with the solvation model density (SMD).[9] Grimme's dispersion with the D3 damping function (GD3) were included for atom-pairwise dispersion corrections.[10] On the basis of the optimized ground-state geometries, time-dependent DFT (TDDFT)[11–13] calculations were performed to compute the singlet–singlet transitions at the same level of theory. The simulated absorption spectra were created using the software Multiwfn.[14] Vibrational frequencies were calculated and all stationary points were verified to be minima on the potential energy surface, with no imaginary frequencies observed (NIMAG = 0). For all the calculations, the Stuttgart effective core potentials (ECPs) and the associated basis set were applied to describe Zn, while the 6-31G(d,p) basis set[15–17] was applied for all other atoms. All the DFT and TDDFT calculations were performed with a pruned (175,974) grid for numerical integration.

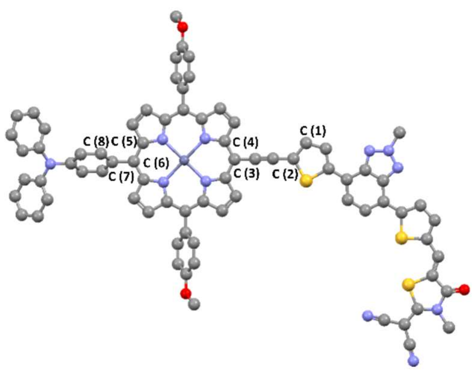

C(1)-C(2)-C(3)-C(4) **0.44°**

C(5)-C(6)-C(7)-C(8) **62.06°**

1'

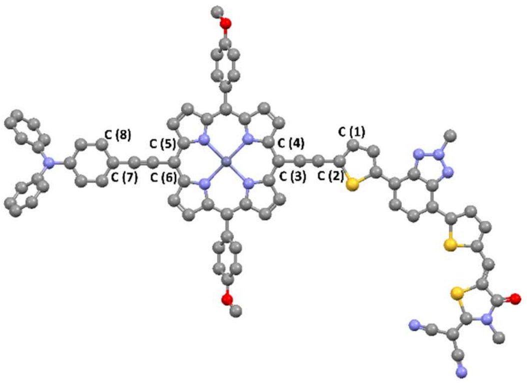

C(1)-C(2)-C(3)-C(4) **1.21°**

C(5)-C(6)-C(7)-C(8) **3.34°**

2'

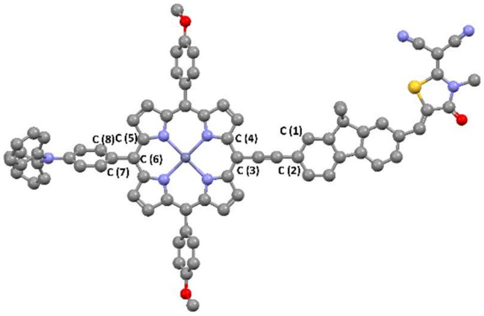

C(1)-C(2)-C(3)-C(4) **3.15°**

C(5)-C(6)-C(7)-C(8) **65.50°**

3'

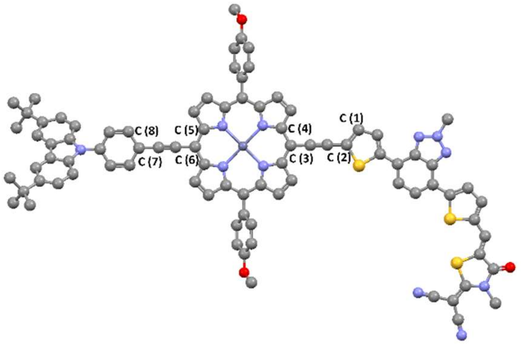

C(1)-C(2)-C(3)-C(4) **4.24°**

C(5)-C(6)-C(7)-C(8) **5.79°**

4'

Figure S4. Optimized ground-state geometries with selected dihedral angles of **1'–4'** computed at the M06 level of theory using toluene as the solvent. All hydrogen atoms are omitted for clarity.

Table S3. The first ten singlet excitations (S_n) of **1'–4'** computed by TDDFT/SMD using toluene as the solvent.

Complex	S_n	Excitation[a] (Coefficient)[b]	Vertical excitation wavelength / nm	f^c
**1'**	S_1	H→L (0.65)	739	1.657
		H→L+1 (0.21)		
	S_2	H→L+1 (0.61)	604	0.158
	S_3	H→L+2 (0.51)	600	0.016
		H−2→L+1 (0.35)		
		H−2→L (0.31)		
	S_4	H−3→L (−0.31)	557	0.359
		H−1→L (0.59)		
	S_5	H–2→L (0.62)	523	0.059
	S_6	H–3→L (0.56)	521	0.650
	S_7	H–1→L+1 (0.63)	483	0.070
	S_8	H→L+3 (0.51)	466	0.601
		H–2→L+2 (–0.32)		
	S_9	H–1→L+2 (0.47)	452	0.392
		H–2→L+1 (0.40)		
		H→L+2 (–0.32)		
	S_{10}	H–3→L+1 (0.43)	442	0.429
		H→L+3 (–0.41)		
**2'**	S_1	H→L (0.63)	780	2.003
		H→L+1 (0.25)		
	S_2	H→L+1 (0.62)	645	0.240
	S_3	H→L+2 (0.52)	619	0.011
		H–2→L+1 (–0.35)		
		H–2→L (–0.30)		
	S_4	H−1→L (0.58)	571	0.507
		H−3→L (–0.32)		
	S_5	H–2→L (0.64)	520	0.156
	S_6	H–3→L (0.49)	516	0.667
		H–1→L+1 (0.33)		
	S_7	H–1→L+1 (0.56)	489	0.023
	S_8	H→L+3 (0.62)	472	0.192
	S_9	H–2→L+1 (0.56)	462	0.780
		H→L+2 (0.39)		

	S_{10}	H–3→L+1 (0.49)	454	0.724
		H–2→L+2 (–0.36)		
**3'**	S_1	H→L (0.60)	653	1.291
		H→L+1 (0.31)		
	S_2	H→L+2 (0.53)	590	0.021
		H−2→L+1 (−0.34)		
	S_3	H→L+1 (0.55)	558	0.081
		H→L (−0.35)		
	S_4	H−1→L (0.61)	516	0.012
		H−1→L+1 (0.32)		
	S_5	H–2→L (0.63)	482	0.207
	S_6	H–1→L+1 (0.47)	465	0.930
	S_7	H–1→L+2 (0.59)	450	0.189
	S_8	H–3→L (0.45)	448	1.019
		H–1→L+1 (0.35)		
	S_9	H–2→L+1 (0.53)	428	1.241
		H–1→L+2 (0.34)		
	S_{10}	H–2→L+2 (0.45)	419	0.442
		H–3→L (0.41)		
**4'**	S_1	H→L (0.63)	756	2.056
	S_2	H→L+1 (0.62)	625	0.157
	S_3	H→L+2 (0.50)	613	0.004
		H–2→L+1 (0.35)		
		H–2→L (–0.33)		
	S_4	H–3→L (0.47)	552	0.613
		H–1→L (0.47)		
	S_5	H–2→L (0.62)	512	0.154
	S_6	H–3→L (0.44)	509	0.379
		H–1→L (0.43)		
	S_7	H–1→L+1 (0.61)	474	0.033
	S_8	H→L+3 (0.50)	464	0.603
		H–2→L+2 (0.32)		
	S_9	H–2→L+1 (0.55)	455	0.946
		H→L+2 (–0.41)		
	S_{10}	H–3→L+1 (0.45)	454	0.385
		H→L+3 (0.41)		

[a] The orbitals involved in the excitation (H = HOMO and L = LUMO).

[b] The coefficients in the configuration interaction (CI) expansion that are less than 0.3 are not listed.

[c] Oscillator strengths.

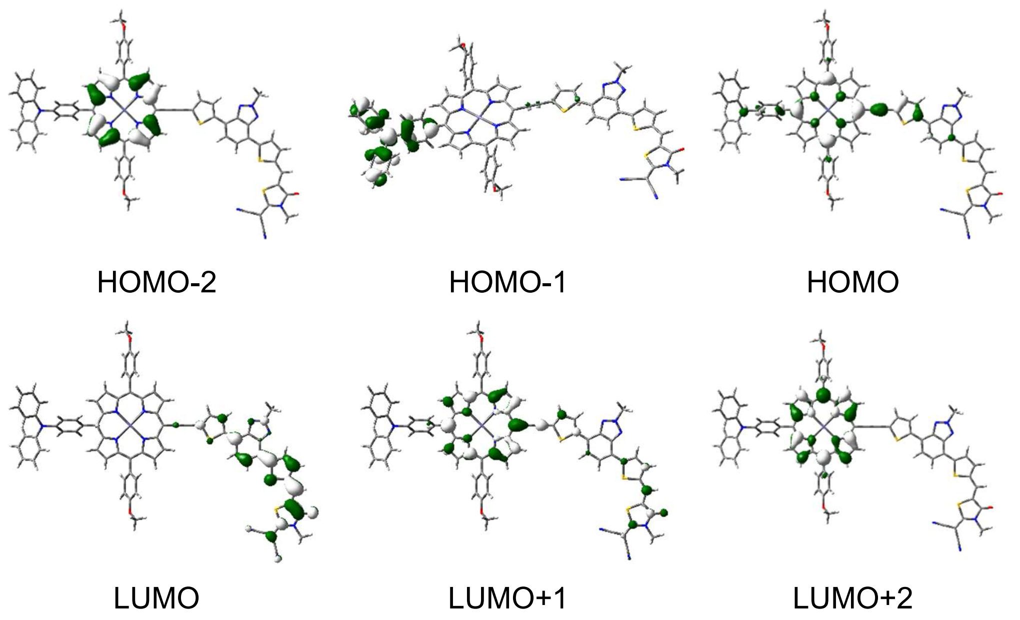

Figure S5. Spatial plots (isovalue = 0.03) of selected molecular orbitals of **1'** at the optimized ground-state geometry.

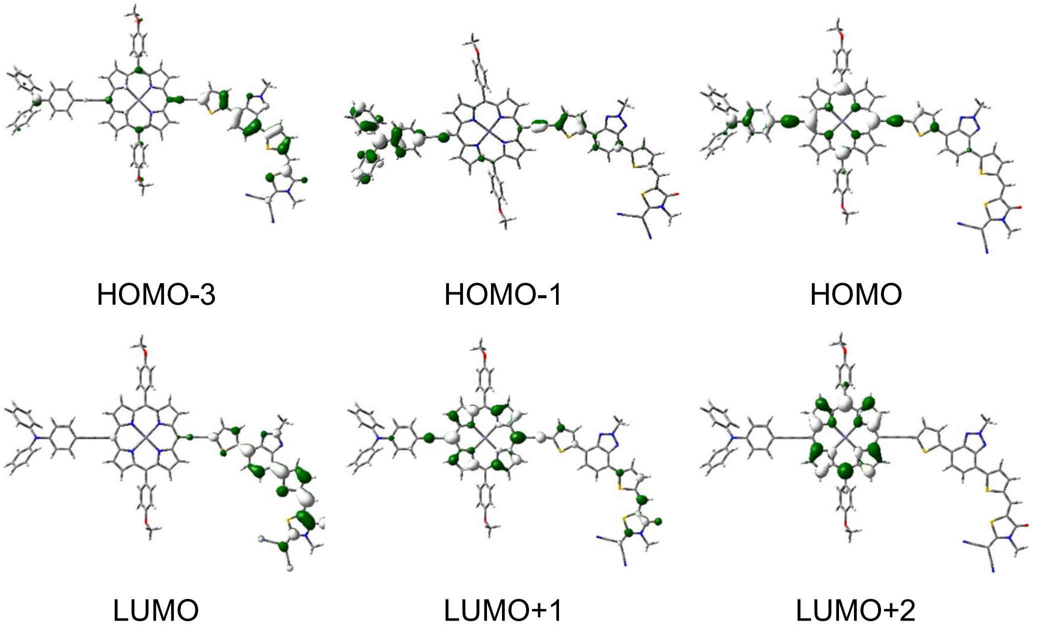

Figure S6. Spatial plots (isovalue = 0.03) of selected molecular orbitals of **2'** at the optimized ground-state geometry.

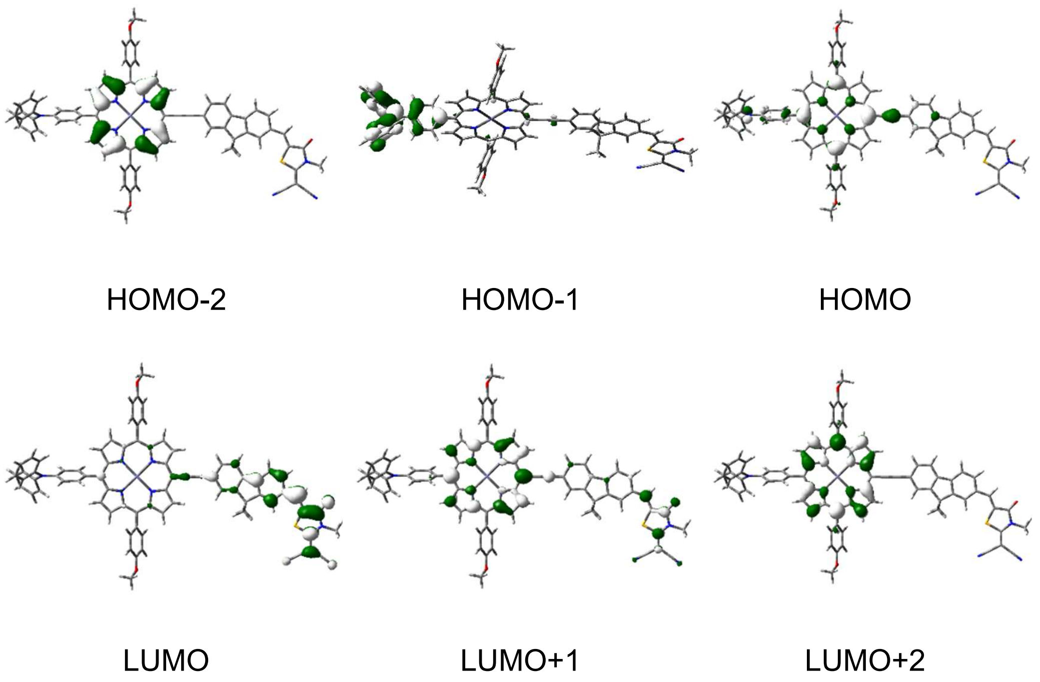

Figure S7. Spatial plots (isovalue = 0.03) of selected molecular orbitals of **3'** at the optimized ground-state geometry.

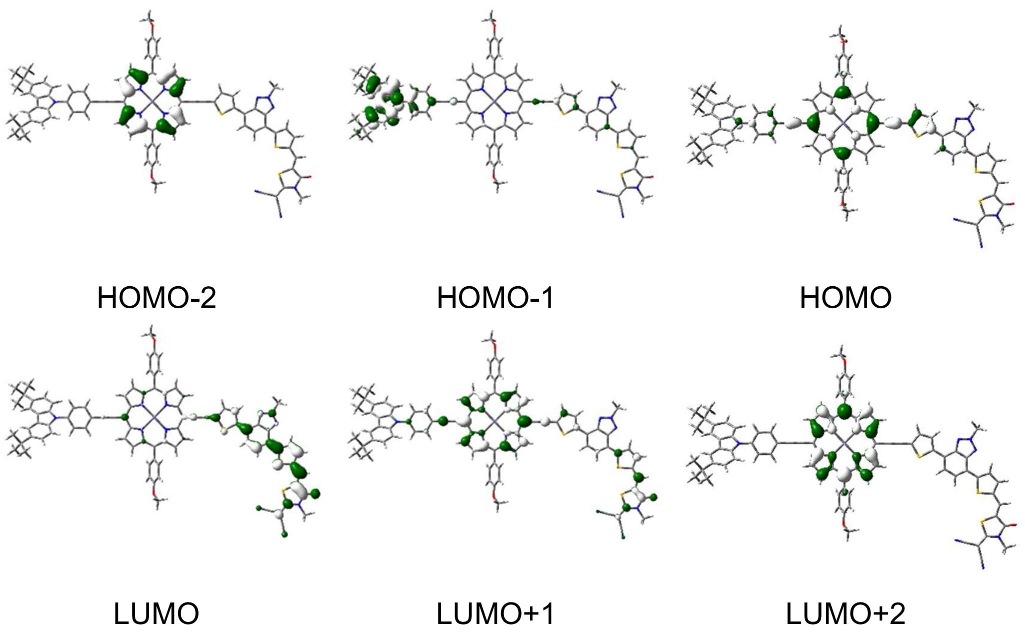

Figure S8. Spatial plots (isovalue = 0.03) of selected molecular orbitals of **4'** at the optimized ground-state geometry.

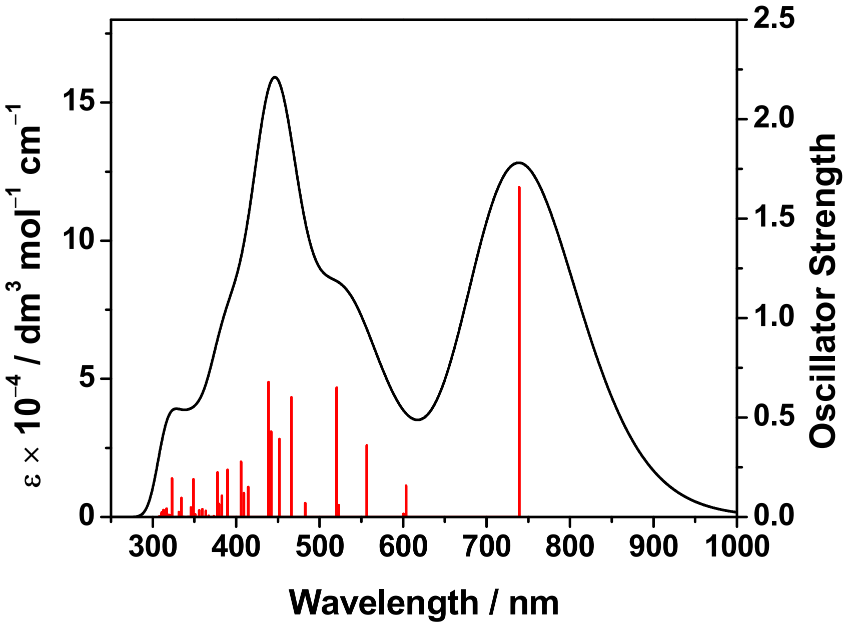

Figure S9. Simulated UV–vis spectrum of **1'** computed by TDDFT/SMD using toluene as the solvent.

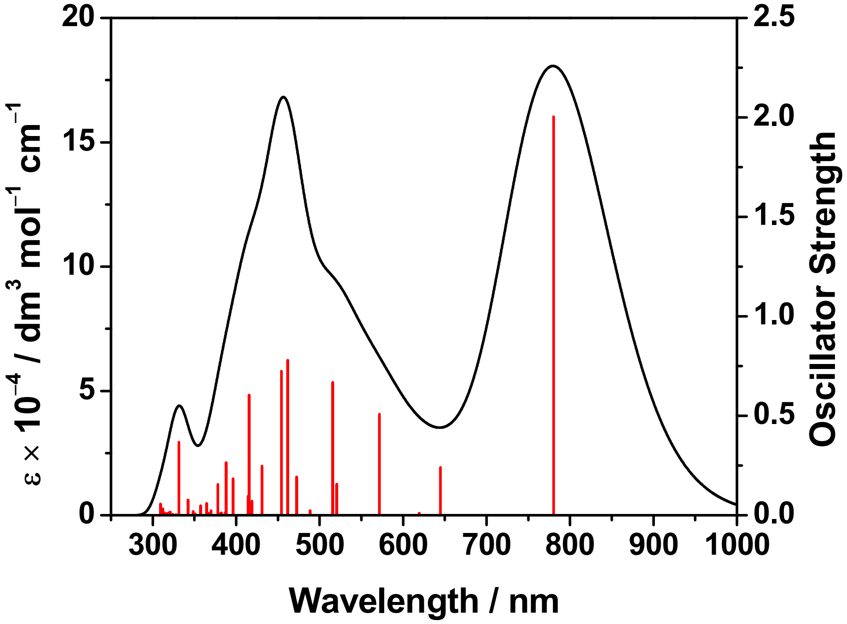

Figure S10. Simulated UV–vis spectrum of **2'** computed by TDDFT/SMD using toluene as the solvent.

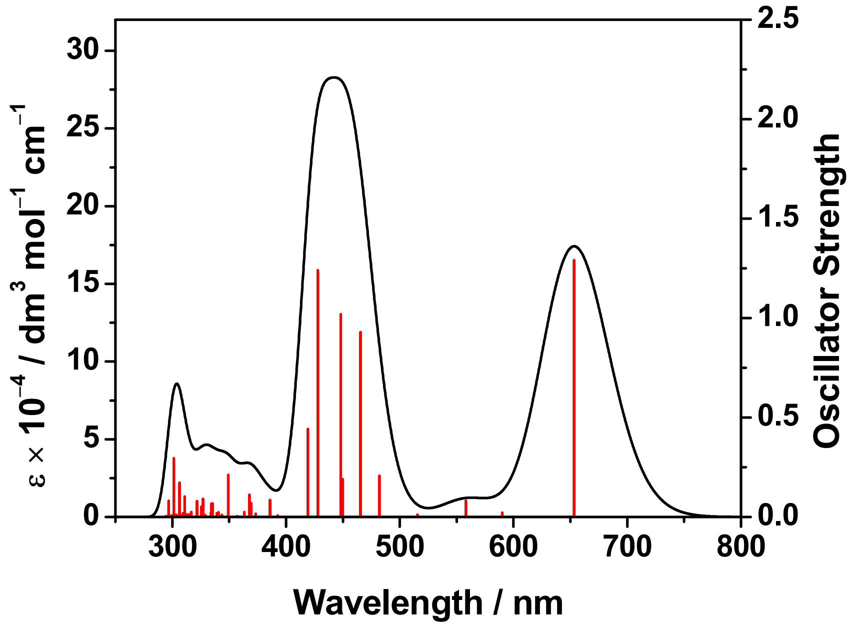

Figure S11. Simulated UV–vis spectrum of **3'** computed by TDDFT/SMD using toluene as the solvent.

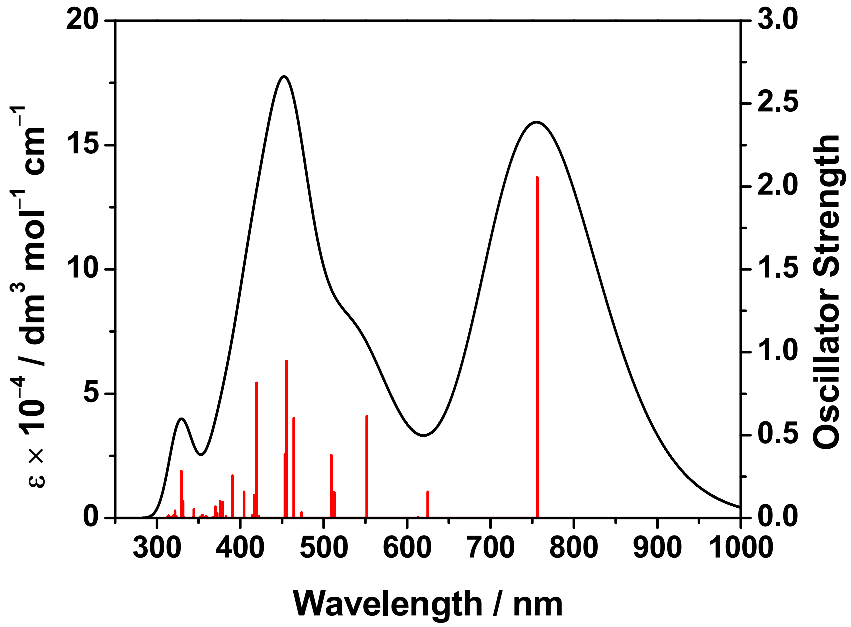

Figure S12. Simulated UV–vis spectrum of **4'** computed by TDDFT/SMD using toluene as the solvent.

Table S4. Cartesian coordinates of the optimized ground-state geometry of **1'**.

Zn	-3.83995	0.627708	-0.04017	C	-12.1397	-6.18745	-0.47
O	-0.82937	-7.96903	-0.02972	H	-11.01	-4.70622	0.614998
N	-4.71465	-1.23716	-0.0702	C	-14.1025	-5.39077	-1.60568
N	-1.97637	-0.25451	-0.0144	H	-14.4884	-3.27473	-1.45308
C	-6.05662	-1.49715	0.012102	C	-13.2561	-6.4414	-1.26216
C	-6.25935	-2.91303	0.171369	H	-11.4744	-7.00058	-0.18758
H	-7.21752	-3.4018	0.294497	H	-14.975	-5.57442	-2.22917
C	-5.02878	-3.49369	0.172508	H	-13.465	-7.45059	-1.60888
H	-4.79595	-4.5438	0.29626	C	-13.5051	-1.72149	0.565846
C	-4.06526	-2.43721	0.01437	C	-13.5822	-0.36967	0.214538
C	-2.66554	-2.62775	-0.01326	C	-14.4878	-2.26888	1.396925
C	-1.70919	-1.60141	-0.04953	C	-14.6186	0.418963	0.697668
C	-0.28362	-1.79998	-0.13619	H	-12.8243	0.05534	-0.44029
H	0.213112	-2.75924	-0.20736	C	-15.5306	-1.47647	1.860076
C	0.291698	-0.56815	-0.131	H	-14.4261	-3.31869	1.675923
H	1.345637	-0.32305	-0.18703	C	-15.6006	-0.1286	1.518778
C	-0.77863	0.388093	-0.05783	H	-14.6653	1.468875	0.416304
C	-0.59869	1.79218	-0.05198	H	-16.2877	-1.91604	2.505964
C	-2.17016	-4.02836	0.007641	H	-16.4144	0.490162	1.889197
C	-2.48219	-4.91976	-1.02971	C	0.734248	2.267175	-0.05894
H	-3.08939	-4.57245	-1.86415	C	1.883449	2.675151	-0.05304
C	-2.02132	-6.22391	-1.0159	H	0.197178	-9.50909	0.763623
H	-2.25317	-6.91491	-1.82288	C	-7.54289	9.753041	-1.2193
C	-1.23143	-6.67995	0.045085	H	-7.78095	10.79007	-0.97124
C	-0.91188	-5.81046	1.088808	H	-6.92544	9.739445	-2.12879
H	-0.30725	-6.1399	1.928808	H	-8.47961	9.211744	-1.41447
C	-1.38206	-4.50028	1.058387	C	3.211435	3.113674	-0.03906
H	-1.13716	-3.82934	1.880365	S	4.524191	1.957717	-0.0293
C	-0.02187	-8.46798	1.01177	C	3.697016	4.406768	-0.02191
H	-0.53998	-8.43639	1.980783	C	5.718081	3.237797	-0.00384
H	0.925417	-7.91633	1.093325	C	5.10048	4.473422	-0.00087
O	-6.85368	9.22377	-0.11069	C	7.131608	2.936088	0.013634
N	-2.96345	2.493369	-0.02544	H	5.667397	5.398309	0.016617
N	-5.7007	1.509948	-0.04796	C	7.634507	1.642456	-0.05739
C	-1.63013	2.760441	-0.04894	C	8.129583	3.950208	0.107889
C	-1.41249	4.180188	-0.1019	C	9.005963	1.343662	-0.03176
H	-0.44215	4.660584	-0.13633	H	6.943764	0.805449	-0.14133
C	-2.64006	4.764052	-0.12111	N	7.992871	5.287307	0.195743
H	-2.86748	5.82102	-0.18093	C	9.5161	3.648299	0.133652
C	-3.61414	3.702029	-0.06532	C	9.999312	2.309225	0.066238
C	-5.00463	3.885416	-0.08358	H	9.285433	0.293012	-0.09518
C	-5.96562	2.850783	-0.07791	N	9.236233	5.713447	0.269939
C	-7.3894	3.057502	-0.09339	N	10.19394	4.807036	0.236132
H	-7.88501	4.019947	-0.10355	C	11.40995	1.994306	0.097891
C	-7.97003	1.826999	-0.09258	C	9.544496	7.127474	0.316851
H	-9.02797	1.599018	-0.1207	S	11.95845	0.337866	0.038254
C	-6.90577	0.85904	-0.048	C	12.48558	2.863568	0.176072
C	-7.08826	-0.53717	-0.01041	H	9.657419	7.518252	-0.69889
C	-8.48334	-1.0456	0.014919	H	8.724638	7.641083	0.820557
C	-8.97104	-1.85283	-1.01962	H	10.47689	7.257732	0.867784
H	-8.32073	-2.08896	-1.86036	C	13.63502	0.833602	0.117081
C	-10.2722	-2.33129	-1.00786	H	12.35028	3.938615	0.22351
H	-10.6375	-2.93852	-1.83306	C	13.72532	2.214054	0.186361
C	-11.1275	-2.03152	0.058974	C	14.73879	-0.05754	0.109029

C	-10.6465	-1.23271	1.103101	H	15.72145	0.416242	0.165395
H	-11.2947	-1.006	1.946714	C	14.78	-1.41092	0.041056
C	-9.34933	-0.74319	1.072198	C	16.05718	-2.12186	0.042941
H	-8.98673	-0.13434	1.89883	S	13.42661	-2.53502	-0.06151
C	-5.50689	5.285404	-0.11506	O	17.16628	-1.63689	0.104745
C	-5.30056	6.148905	0.970061	N	15.83138	-3.51657	-0.04205
H	-4.77403	5.780672	1.84926	C	16.97563	-4.41896	-0.06115
C	-5.76049	7.453769	0.943638	H	16.99226	-5.00165	-0.9855
H	-5.60767	8.125309	1.785158	H	17.86596	-3.78956	-0.01146
C	-6.444	7.935867	-0.17745	H	16.95478	-5.08901	0.802023
C	-6.65974	7.092413	-1.26811	C	14.53407	-3.90267	-0.10255
H	-7.18198	7.442562	-2.15382	C	14.02511	-5.18833	-0.18744
C	-6.19081	5.781848	-1.22495	C	12.61301	-5.34658	-0.23857
H	-6.35495	5.130255	-2.08203	N	11.45229	-5.43988	-0.27816
N	-12.4473	-2.52889	0.081966	C	14.80168	-6.37683	-0.22588
C	-12.7178	-3.84357	-0.37082	N	15.40342	-7.37398	-0.25951
C	-11.8738	-4.90144	-0.01716	H	3.040027	5.270894	-0.02367
C	-13.8347	-4.09814	-1.17292	H	14.68331	2.723869	0.242333

Table S5. Cartesian coordinates of the optimized ground-state geometry of **2'**.

Zn	-3.1999	0.930206	0.092749	C	8.891541	3.750233	0.179332
O	-0.49412	-7.77005	-0.08318	C	9.652332	1.109465	0.00927
N	-4.14387	-0.90192	0.056523	H	7.567143	0.660606	-0.06247
N	-1.3741	-0.02753	0.111003	N	8.81396	5.091303	0.277186
C	-5.4897	-1.11458	0.084614	C	10.264	3.388942	0.178224
C	-5.76181	-2.52283	0.131812	C	10.68806	2.03097	0.09166
H	-6.75001	-2.96487	0.170326	H	9.884868	0.048042	-0.06244
C	-4.55678	-3.15563	0.138153	N	10.07554	5.4633	0.331642
H	-4.37151	-4.22107	0.190765	N	10.99263	4.517025	0.275126
C	-3.54409	-2.1335	0.079764	C	12.08476	1.657478	0.086328
C	-2.15514	-2.37408	0.067402	C	10.44434	6.862639	0.382173
C	-1.15867	-1.38234	0.064841	S	12.56512	-0.01673	-0.03932
C	0.258115	-1.6351	-0.01049	C	13.19573	2.480611	0.168674
H	0.718197	-2.61236	-0.08345	H	10.56926	7.252057	-0.63267
C	0.880649	-0.42587	0.007412	H	9.648847	7.408047	0.891407
H	1.943725	-0.22177	-0.0389	H	11.38372	6.951797	0.92942
C	-0.15228	0.569981	0.078418	C	14.26132	0.410041	0.019785
C	0.079523	1.966754	0.088278	H	13.10497	3.558085	0.252704
C	-1.70884	-3.79179	0.053263	C	14.40823	1.782823	0.131304
C	-2.01062	-4.63327	-1.02746	C	15.32913	-0.52185	-0.04404
H	-2.57488	-4.23566	-1.86957	H	16.33066	-0.08752	-0.00215
C	-1.59266	-5.95194	-1.04554	C	15.31756	-1.87319	-0.15188
H	-1.81657	-6.60546	-1.88532	C	16.56765	-2.62863	-0.20975
C	-0.85747	-6.47188	0.025221	S	13.9219	-2.94541	-0.24308
C	-0.54853	-5.65162	1.111153	O	17.69475	-2.18454	-0.17214
H	0.014801	-6.03063	1.958846	N	16.28927	-4.01163	-0.32304
C	-0.97485	-4.32615	1.112792	C	17.39922	-4.95259	-0.40472
H	-0.73698	-3.69283	1.966142	H	17.36498	-5.50906	-1.34465
C	0.258481	-8.33281	0.966917	H	18.31281	-4.35656	-0.36787
H	-0.29304	-8.31818	1.917718	H	17.38244	-5.64554	0.440264
H	1.219627	-7.81671	1.101805	C	14.97758	-4.34997	-0.35212
O	-5.90138	9.632802	0.265401	C	14.42063	-5.61442	-0.45203
N	-2.2561	2.762921	0.12842	C	13.00291	-5.72191	-0.46604
N	-5.02563	1.888354	0.075203	N	11.83894	-5.77373	-0.47456
C	-0.91276	2.976225	0.097533	C	15.15277	-6.82823	-0.54014

C	-0.63939	4.385519	0.052156	N	15.71736	-7.84479	-0.61353
H	0.34866	4.827885	0.012617	H	3.863557	5.291696	0.138506
C	-1.8437	5.018191	0.049705	H	15.3871	2.251849	0.182581
H	-2.02881	6.083827	-0.00036	C	-7.83505	-0.53301	0.08601
C	-2.85793	3.996146	0.107833	C	-8.99765	-0.89591	0.066954
C	-4.24317	4.23525	0.119982	C	-10.3501	-1.31759	0.032638
C	-5.24191	3.240623	0.120965	C	-11.399	-0.38376	0.082689
C	-6.65762	3.494427	0.19153	C	-10.6791	-2.68051	-0.06131
H	-7.11734	4.472011	0.262884	H	-11.1634	0.675175	0.167062
C	-7.28047	2.284381	0.170464	C	-12.7187	-0.79362	0.043359
H	-8.34302	2.077973	0.212266	C	-11.9979	-3.09237	-0.11364
C	-6.24912	1.288872	0.102524	H	-9.87958	-3.4169	-0.11134
C	-6.48286	-0.10675	0.089636	H	-13.5168	-0.05723	0.098269
C	-4.69134	5.652587	0.134253	C	-13.0411	-2.15535	-0.06131
C	-4.39687	6.492362	1.21813	H	-12.2318	-4.15028	-0.20496
H	-3.83873	6.093067	2.063519	N	-14.3818	-2.57009	-0.11517
C	-4.8132	7.811752	1.234544	C	-15.3595	-1.76347	-0.75331
H	-4.59402	8.46429	2.076347	C	-14.7792	-3.80717	0.45442
C	-5.53938	8.333695	0.15884	C	-15.1008	-1.18999	-2.00191
C	-5.84187	7.514797	-0.92995	C	-16.598	-1.54949	-0.14201
H	-6.39812	7.895484	-1.78158	C	-14.3337	-4.17804	1.726753
C	-5.41743	6.188717	-0.92977	C	-15.6345	-4.65967	-0.24961
H	-5.65005	5.556406	-1.78533	C	-16.0648	-0.40275	-2.61915
C	1.429772	2.38906	0.079449	H	-14.1408	-1.36694	-2.48255
C	2.594991	2.748886	0.082051	C	-17.5626	-0.7755	-0.77508
H	0.452376	-9.37122	0.688465	H	-16.7958	-1.99527	0.830717
C	-6.63928	10.19899	-0.79288	C	-14.7292	-5.39109	2.27627
H	-6.83152	11.23835	-0.51664	H	-13.6774	-3.50869	2.279137
H	-6.0773	10.1819	-1.73752	C	-16.0392	-5.8624	0.315647

Table S6. Cartesian coordinates of the optimized ground-state geometry of **3'**.

Zn	-2.67669	0.436455	0.017108	H	-7.5359	-1.43956	1.94318
O	-4.47683	9.366958	0.045712	H	-10.2746	-0.16228	-1.9801
N	-4.38483	1.587639	0.010147	C	-10.2811	-1.08316	-0.03292
N	-1.53094	2.151535	-0.03591	H	-9.95579	-1.9411	1.915792
C	-5.67285	1.122723	0.0157	N	-11.6657	-1.36138	-0.04677
C	-6.5822	2.238567	0.020737	C	-12.2801	-1.82982	-1.23336
H	-7.66256	2.169627	0.040414	C	-12.4393	-1.16723	1.12257
C	-5.82524	3.368857	-0.00457	C	-11.6472	-2.79655	-2.02175
H	-6.17277	4.394049	-0.02584	C	-13.5257	-1.33266	-1.63036
C	-4.44722	2.95293	0.000658	C	-12.2297	-0.045	1.931174
C	-3.34282	3.832542	-0.01306	C	-13.4238	-2.09361	1.482534
C	-1.99451	3.443495	-0.0185	C	-12.2481	-3.2475	-3.19008
C	-0.87272	4.348995	0.027444	H	-10.682	-3.1904	-1.71015
H	-0.94058	5.428776	0.070583	C	-14.1271	-1.80347	-2.79069
C	0.253842	3.58808	0.025989	H	-14.0164	-0.57531	-1.0226
H	1.284876	3.918581	0.059775	C	-12.9849	0.137158	3.082677
C	-0.17224	2.215371	-0.00821	H	-11.4711	0.682172	1.649103
C	0.70309	1.104404	0.010867	C	-14.1867	-1.89268	2.625921
C	-3.63264	5.291512	-0.02045	H	-13.5846	-2.97066	0.859187
C	-4.24809	5.913762	1.075076	C	-13.4921	-2.75873	-3.5797
H	-4.51162	5.31664	1.946768	H	-11.7435	-3.99941	-3.79313
C	-4.516	7.271274	1.069019	H	-15.0963	-1.40765	-3.08696
H	-4.9872	7.759288	1.918912	C	-13.9701	-0.78098	3.435528
C	-4.17477	8.050891	-0.04134	H	-12.8092	1.014588	3.701475

C	-3.56146	7.45081	-1.1417	H	-14.9488	-2.62186	2.892498
H	-3.2908	8.030419	-2.01947	H	-13.963	-3.11941	-4.491
C	-3.29753	6.083604	-1.11868	H	-14.5642	-0.6312	4.333777
H	-2.82413	5.618868	-1.98239	C	4.679482	1.889321	0.070025
C	-4.15686	10.18897	-1.05274	C	5.609655	0.832483	0.038766
H	-4.68583	9.876218	-1.96434	C	5.137624	3.221516	0.124563
H	-3.07591	10.20133	-1.25249	C	6.959369	1.121527	0.063857
O	-0.87048	-8.49246	-0.01184	H	5.249259	-0.19497	-0.00513
N	-0.96548	-0.71507	-0.00524	C	6.492406	3.508209	0.150143
N	-3.81999	-1.27788	0.085067	H	4.404965	4.025202	0.147976
C	0.314992	-0.25624	0.027661	C	8.127768	0.155364	0.037463
C	1.230245	-1.36167	0.107697	C	7.405332	2.454018	0.120777
H	2.308865	-1.27419	0.156164	H	6.832709	4.541151	0.193407
C	0.477833	-2.49386	0.129401	C	9.301192	1.114256	0.09055
H	0.826299	-3.51599	0.20791	C	8.859576	2.450712	0.139471
C	-0.90297	-2.08548	0.049005	C	10.64634	0.817122	0.095161
C	-2.00232	-2.95862	0.044099	C	9.779668	3.497049	0.197477
C	-3.35842	-2.56324	0.027796	C	11.59256	1.861924	0.150689
C	-4.47262	-3.46697	-0.08886	H	10.9565	-0.22608	0.062834
H	-4.4015	-4.54317	-0.18327	C	11.13024	3.193381	0.204049
C	-5.60208	-2.70806	-0.09232	H	9.448375	4.5326	0.238058
H	-6.62531	-3.04908	-0.18863	H	11.86513	3.995355	0.249414
C	-5.18637	-1.33484	0.02044	C	8.140522	-0.66604	-1.254
C	-6.06057	-0.23062	0.015547	H	7.251263	-1.30584	-1.31314
C	-1.71866	-4.41736	0.050467	H	8.154187	-0.0175	-2.13782
C	-1.02839	-5.02615	-1.00796	H	9.02289	-1.31733	-1.29204
H	-0.7064	-4.41806	-1.85186	C	8.11132	-0.77213	1.254669
C	-0.76184	-6.38376	-1.00208	H	8.105492	-0.20088	2.19037
H	-0.23341	-6.86057	-1.82419	H	7.220627	-1.41257	1.239532
C	-1.17908	-7.17811	0.071188	H	8.992037	-1.42678	1.256969
C	-1.86868	-6.59249	1.133921	C	13.01909	1.663155	0.156981
H	-2.19922	-7.18372	1.983009	H	13.62239	2.57058	0.235459
C	-2.13079	-5.22538	1.111132	C	13.77278	0.543173	0.077084
H	-2.66207	-4.77262	1.946985	C	15.23707	0.63655	0.104282
C	2.094147	1.379219	0.03186	S	13.25849	-1.13607	-0.07813
C	3.288541	1.616983	0.048392	O	15.9035	1.642961	0.203053
H	-4.47733	11.19952	-0.78889	N	15.80819	-0.65292	-0.00451
C	-1.26929	-9.33051	1.048197	C	14.93157	-1.68049	-0.11066
H	-0.92984	-10.3371	0.79337	C	17.25926	-0.79058	0.01117
H	-0.80828	-9.03171	2.000382	C	15.20494	-3.03207	-0.23498
H	-2.36191	-9.34506	1.168078	H	17.61632	-1.23117	-0.92298
C	-7.5182	-0.52306	-0.00345	H	17.66535	0.216959	0.116014
C	-8.31143	-0.17738	-1.1028	H	17.57862	-1.40232	0.858664
C	-8.13609	-1.15826	1.079254	C	14.10472	-3.92865	-0.32514
H	-7.85023	0.315669	-1.9571	C	16.50316	-3.60636	-0.28444
C	-9.67246	-0.44509	-1.11942	N	13.17869	-4.63217	-0.3956
C	-9.49393	-1.44126	1.067183	N	17.55075	-4.11417	-0.32914

Table S7. Cartesian coordinates of the optimized groun-state geometry of **4'**.

Zn	1.581173	1.343532	-0.32191	S	-14.1282	-0.46622	0.227704
O	-0.51351	-7.52172	-0.05765	C	-14.8945	1.989959	-0.00699
N	2.649325	-0.41767	-0.24598	H	-12.5297	6.907537	0.325743
N	-0.17383	0.260013	-0.34758	H	-11.6038	7.028645	-1.19858
C	4.006209	-0.53639	-0.24961	H	-13.3132	6.484054	-1.22339

C	4.376306	-1.9236	-0.27241	C	-15.8443	-0.12467	0.261948
H	5.392968	-2.29686	-0.29001	H	-14.8631	3.067626	-0.12516
C	3.218796	-2.63847	-0.2888	C	-16.0663	1.235394	0.122573
H	3.108891	-3.71477	-0.32941	C	-16.8565	-1.10702	0.418325
C	2.13613	-1.68855	-0.26041	H	-17.88	-0.72568	0.431212
C	0.768379	-2.02534	-0.26316	C	-16.7639	-2.45218	0.55857
C	-0.29558	-1.1048	-0.28488	C	-17.9625	-3.27388	0.719173
C	-1.69182	-1.4546	-0.2174	S	-15.3067	-3.44285	0.57983
H	-2.08359	-2.46055	-0.13612	O	-19.113	-2.89405	0.747779
C	-2.39653	-0.29152	-0.2543	N	-17.5986	-4.63588	0.84576
H	-3.47151	-0.16088	-0.21827	C	-18.6447	-5.63425	1.028543
C	-1.4346	0.772284	-0.33059	H	-18.516	-6.158	1.979118
C	-1.76202	2.149292	-0.35992	H	-19.5915	-5.09153	1.037225
C	0.420886	-3.47024	-0.23442	H	-18.6437	-6.35152	0.203921
C	0.766195	-4.27431	0.861655	C	-16.2708	-4.90024	0.792252
H	1.289979	-3.8272	1.70508	C	-15.6357	-6.12812	0.881169
C	0.4411	-5.61857	0.89296	C	-14.2163	-6.15338	0.800103
H	0.698925	-6.24375	1.744478	N	-13.0534	-6.13692	0.729896
C	-0.24149	-6.20276	-0.17952	C	-16.2875	-7.37947	1.042916
C	-0.59341	-5.42005	-1.28015	N	-16.7856	-8.42453	1.174446
H	-1.1182	-5.84807	-2.12926	H	-5.71806	5.237094	-0.54205
C	-0.26093	-4.0681	-1.29491	H	-17.0689	1.654742	0.119277
H	-0.53173	-3.46407	-2.15963	C	6.304214	0.204413	-0.21876
C	-1.20632	-8.15153	-1.11091	C	7.484824	-0.08827	-0.17068
H	-0.64146	-8.11037	-2.0531	C	8.858274	-0.43736	-0.09902
H	-2.19968	-7.70803	-1.26828	C	9.855939	0.545221	-0.20791
O	3.670313	10.20838	-0.65238	C	9.245085	-1.77423	0.092023
N	0.51283	3.105398	-0.39752	H	9.566003	1.581589	-0.36635
N	3.335799	2.427506	-0.29579	C	11.19541	0.201768	-0.13444
C	-0.84256	3.225407	-0.37991	C	10.58361	-2.11516	0.184629
C	-1.21289	4.612729	-0.36122	H	8.478882	-2.54041	0.18776
H	-2.22925	4.986638	-0.33607	H	11.96551	0.960989	-0.24984
C	-0.05506	5.327216	-0.36261	C	11.5685	-1.1299	0.069018
H	0.055583	6.403776	-0.33074	H	10.87744	-3.14608	0.367775
C	1.027473	4.376906	-0.39432	N	12.93108	-1.47785	0.16153
C	2.39398	4.712312	-0.39743	C	13.56344	-2.50972	-0.53634
C	3.458605	3.790753	-0.36608	C	13.87723	-0.86993	0.990737
C	4.855052	4.140386	-0.4234	C	13.06268	-3.37542	-1.50092
H	5.247213	5.145911	-0.50834	C	14.92065	-2.56077	-0.15478
C	5.559287	2.97738	-0.36938	C	13.73147	0.165908	1.905174
H	6.634063	2.844105	-0.39303	C	15.12072	-1.51462	0.821796
C	4.597427	1.913876	-0.29576	C	13.93374	-4.3072	-2.05613
C	4.925827	0.538553	-0.25455	H	12.02531	-3.32843	-1.82365
C	2.741735	6.157032	-0.43896	C	15.76724	-3.5042	-0.72837
C	2.400626	6.949271	-1.5448	C	14.85281	0.560497	2.627771
H	1.880952	6.492667	-2.38568	H	12.77242	0.653045	2.064414
C	2.723699	8.293783	-1.58824	C	16.22433	-1.09657	1.558825
H	2.468632	8.909934	-2.44716	C	15.28648	-4.39946	-1.68613
C	3.399724	8.889926	-0.51833	H	13.53508	-4.98362	-2.80758
C	3.74721	8.119191	0.592077	H	16.81327	-3.53373	-0.42304
H	4.266246	8.556918	1.439838	C	16.11067	-0.04664	2.472392
C	3.41689	6.76681	0.618916	H	14.73274	1.372254	3.340346
H	3.683813	6.172054	1.491241	H	17.17943	-1.60303	1.418971
C	-3.13771	2.480434	-0.35634	C	17.3406	0.393782	3.266945
C	-4.32183	2.771581	-0.35767	C	16.23692	-5.43216	-2.29314
H	-1.32979	-9.19724	-0.82002	C	15.54685	-6.32344	-3.32315
C	4.353207	10.85052	0.399524	H	14.71994	-6.89559	-2.88301
H	4.476023	11.8937	0.099261	H	15.15375	-5.74526	-4.16932

H	3.781525	10.81719	1.337916	H	16.26727	-7.04622	-3.7266
H	5.346594	10.41138	0.56904	C	16.78978	-6.33211	-1.18238
C	-5.68563	3.081183	-0.35646	H	17.47532	-7.08165	-1.60078
S	-6.88309	1.813395	-0.22326	H	17.34427	-5.76159	-0.42753
C	-6.29064	4.319349	-0.45131	H	15.97892	-6.86399	-0.66812
C	-8.1916	2.973376	-0.29559	C	17.39756	-4.71385	-2.99022
C	-7.69393	4.25625	-0.41805	H	17.97482	-4.09168	-2.29573
C	-9.57058	2.544916	-0.22154	H	18.08902	-5.44313	-3.43385
H	-8.34566	5.121406	-0.48037	H	17.03012	-4.06267	-3.7938
C	-9.94655	1.219657	-0.03976	C	17.85747	-0.78247	4.102618
C	-10.6619	3.456711	-0.32464	H	18.1438	-1.63688	3.477555
C	-11.2838	0.799288	0.038691	H	18.74239	-0.48345	4.680812
H	-9.17811	0.454633	0.055214	H	17.09187	-1.12856	4.808922
N	-10.6549	4.790695	-0.51041	C	18.43796	0.850172	2.299229
C	-12.0139	3.031921	-0.24585	H	18.09591	1.695227	1.687884
C	-12.3661	1.663913	-0.05763	H	19.32991	1.171852	2.854003
H	-11.4608	-0.26456	0.189116	H	18.74442	0.048394	1.616671
N	-11.9347	5.098253	-0.53789	C	17.03663	1.550523	4.21612
N	-12.8009	4.115456	-0.38495	H	16.68825	2.442055	3.678782
C	-13.7408	1.2248	0.032442	H	16.27924	1.280784	4.963399
C	-12.3754	6.471724	-0.66592	H	17.94789	1.830499	4.759654

Memory Properties

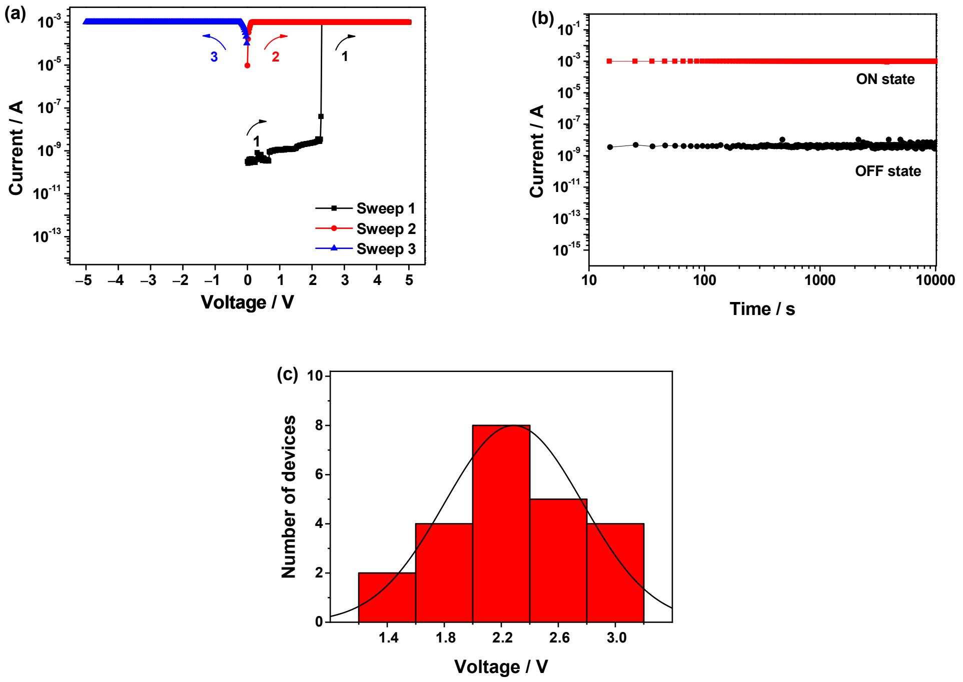

Figure S13. (a) Representative current–voltage characteristics of an ITO/LiF/**2**/Al device. (b) Retention time of the memory devices fabricated with **2** in "OFF"and "ON" states under constant stress (1.0 V). (c) Bar chart of the number of devices against threshold voltage of "ON" state among 23 devices.

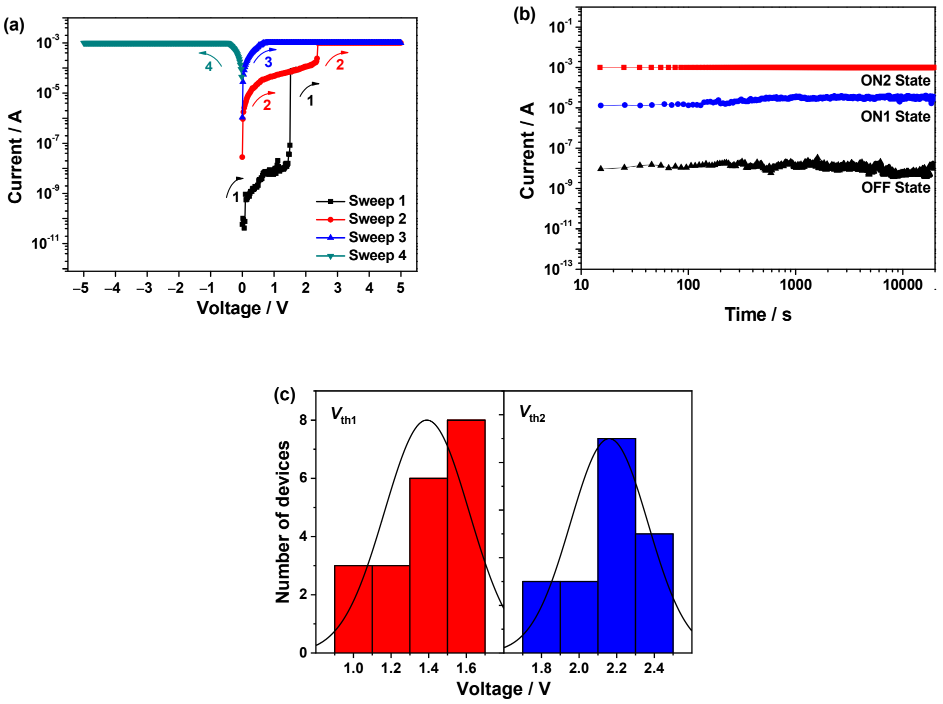

Figure S14. (a) Representative current–voltage characteristics of an ITO/LiF/**4**/Al device. (b) Retention time of the memory devices fabricated with **4** in “OFF”, “ON1” and”ON2” states under constant stress (1.0 V). (c) Bar charts of the number of devices against threshold voltage of “ON” state among 20 devices.

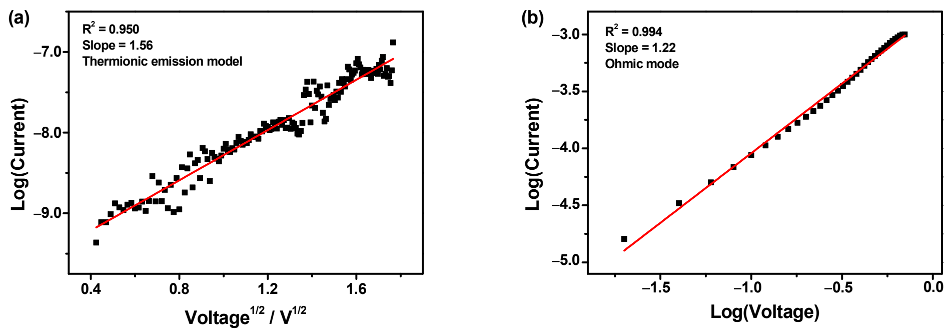

Figure S15. (a) Plot of log(I) *vs*. $V^{1/2}$ obtained by fitting the I–V characteristics of the "OFF" state (from 0.18 to 3.12 V) and (b) plot of log(I) *vs*. log(V) obtained by fitting the I–V characteristics of the "ON" state (from 0.02 to 0.7 V) of the memory device fabricated with **1**.

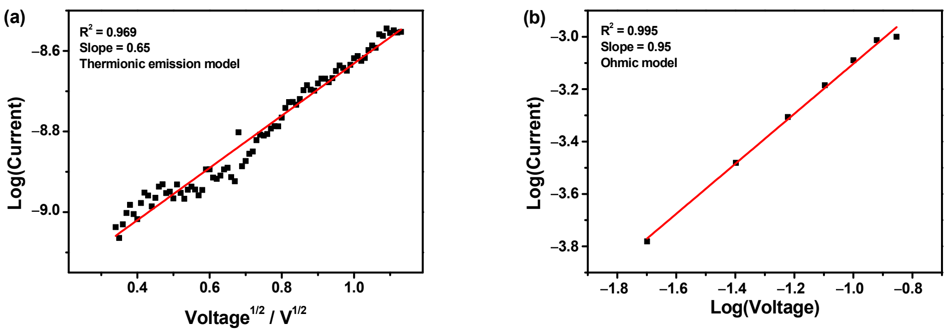

Figure S16. (a) Plot of log(I) *vs*. $V^{1/2}$ obtained by fitting the I–V characteristics of the "OFF" state (from 0.68 to 2.26 V) and (b) plot of log(I) *vs*. log(V) obtained by fitting the I–V characteristics of the "ON" state (from 0.02 to 0.14 V) of the memory device fabricated with **2**.

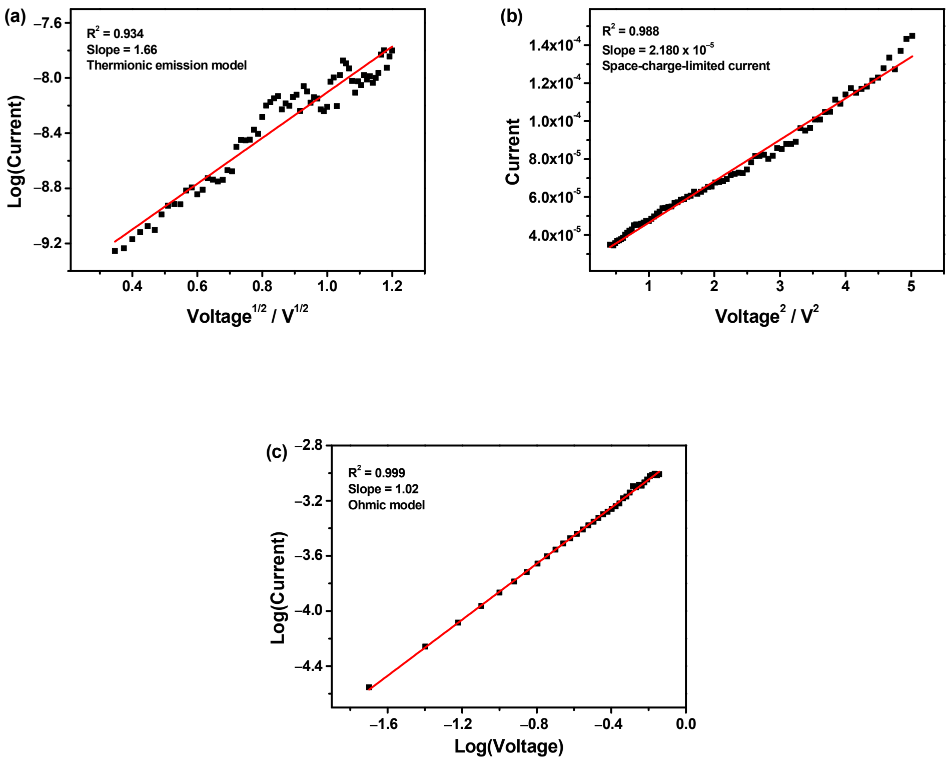

Figure S17. (a) Plot of log(I) *vs*. $V^{1/2}$ obtained by fitting the I–V characteristics of the "OFF" state (from 0.12 to 1.44 V). (b) Plot of I *vs*. V^2 obtained by fitting the I–V characteristics of the "ON1" state (from 0.64 to 2.44 V). (c) Plot of log(I) *vs*. log(V) obtained by fitting the I–V characteristics of the "ON2" state (from 0.02 to 0.72 V) of the memory device fabricated with **4**.

References

1. Crosby, G. A.; Demas, J. N. "Measurement of photoluminescence quantum yields. Review", *J. Phys. Chem.*, **1971**, *75*, 991.
2. Melhuish, W. H. "Quantum efficiencies of fluorescence of organic substances: effect of solvent and concentration of the fluorescent solute1", *J. Phys. Chem.*, **1961**, *65*, 229.
3. Mathew, S.; Yella, A.; Gao, P.; Humphry-Baker, R.; Curchod, B. F. E.; Ashari-Astani, N.; Tavernelli, I.; Rothlisberger, U.; Nazeeruddin, M. K.; Grätzel, M. "Dye-Sensitized Solar Cells with 13% Efficiency Achieved Through The Molecular Engineering of Porphyrin Sensitizers", *Nat. Chem.*, **2014**, *6*, 242.
4. Poon, C.-T.; Wu, D.; Lam, W. H.; Yam, V. W.-W. "A Solution-Processable Donor–Acceptor Compound Containing Boron(III) Centers for Small-Molecule-Based High-Performance Ternary Electronic Memory Devices", *Angew. Chem. Int. Ed.*, **2015**, *54*, 10569.
5. Poon, C.-T.; Wu, D.; Yam, V. W.-W. "Boron(III)-Containing Donor–Acceptor Compound with Goldlike Reflective Behavior for Organic Resistive Memory Devices", *Angew. Chem. Int. Ed.*, **2016**, *55*, 3647.
6. Li, P.; Liang, Q.; Hong, E. Y.-H.; Chan, C.-Y.; Cheng, Y.-H.; Leung, M.-Y.; Chan, M.-Y.; Low, K.-H.; Wu, H.; Yam, V. W.-W. "Boron(III) β-Diketonate-Based Small Molecules for Functional Non-Fullerene Polymer Solar Cells and Organic Resistive Memory Devices", *Chem. Sci.*, **2020**, *11*, 11601.
7. Frisch, M. J.; Trucks, G. W.; Schlegel, H. B.; Scuseria, G. E.; Robb, M. A.; Cheeseman, J. R.; Scalmani, G.; Barone, V.; Petersson, G. A.; Nakatsuji, H.; Li, X.; Caricato, M.; Marenich, A. V.; Bloino, J.; Janesko, B. G.; Gomperts, R.; Mennucci, B.; Hratchian, H. P.; Ortiz, J. V.; Izmaylov, A. F.; Sonnenberg, J. L.; Williams; Ding, F.; Lipparini, F.; Egidi, F.; Goings, J.; Peng, B.; Petrone, A.; Henderson, T.; Ranasinghe, D.; Zakrzewski, V. G.; Gao, J.; Rega, N.; Zheng, G.; Liang, W.; Hada, M.; Ehara, M.; Toyota, K.; Fukuda, R.; Hasegawa, J.; Ishida, M.; Nakajima, T.; Honda, Y.; Kitao, O.; Nakai, H.; Vreven, T.; Throssell, K.; Montgomery Jr., J. A.; Peralta, J. E.; Ogliaro, F.; Bearpark, M. J.; Heyd, J. J.; Brothers, E. N.; Kudin, K. N.; Staroverov, V. N.; Keith, T. A.; Kobayashi, R.; Normand, J.; Raghavachari, K.; Rendell, A. P.; Burant, J. C.; Iyengar, S. S.; Tomasi, J.; Cossi, M.; Millam, J. M.; Klene, M.; Adamo, C.; Cammi, R.; Ochterski, J. W.; Martin, R. L.; Morokuma, K.; Farkas, O.; Foresman, J. B.; Fox, D. J. Gaussian 16 Rev. C.01, Wallingford, CT, 2016.
8. Zhao, Y.; Truhlar, D. G. "The M06 suite of density functionals for main group thermochemistry, thermochemical kinetics, noncovalent interactions, excited states, and transition elements: two new functionals and systematic testing of

four M06-class functionals and 12 other functionals", *Theor. Chem. Acc.*, **2008**, *120*, 215.

9. Marenich, A. V.; Cramer, C. J.; Truhlar, D. G. "Universal Solvation Model Based on Solute Electron Density and on a Continuum Model of the Solvent Defined by the Bulk Dielectric Constant and Atomic Surface Tensions", *J. Phys. Chem. B*, **2009**, *113*, 6378.
10. Grimme, S.; Antony, J.; Ehrlich, S.; Krieg, H. "A consistent and accurate ab initio parametrization of density functional dispersion correction (DFT-D) for the 94 elements H-Pu", *J. Chem. Phys.*, **2010**, *132*, 154104.
11. Bauernschmitt, R.; Ahlrichs, R. "Treatment of electronic excitations within the adiabatic approximation of time dependent density functional theory", *Chem. Phys. Lett.*, **1996**, *256*, 454.
12. Casida, M. E.; Jamorski, C.; Casida, K. C.; Salahub, D. R. "Molecular Excitation Energies to High-Lying Bound States from Time-Dependent Density-Functional Response Theory: Characterization and Correction of the Time-Dependent Local Density Approximation Ionization Threshold", *J. Chem. Phys.*, **1998**, *108*, 4439.
13. Stratmann, R. E.; Scuseria, G. E.; Frisch, M. J. "An Efficient Implementation of Time-Dependent Density-Functional Theory for the Calculation of Excitation Energies of Large Molecules", *J. Chem. Phys.*, **1998**, *109*, 8218.
14. Lu, T.; Chen, F. "Multiwfn: A multifunctional wavefunction analyzer", *J. Comput. Chem.*, **2012**, *33*, 580.
15. Hehre, W. J.; Ditchfield, R.; Pople, J. A. "Self-Consistent Molecular Orbital Methods. XII. Further Extensions of Gaussian-Type Basis Sets for Use in Molecular Orbital Studies of Organic Molecules", *J. Chem. Phys.*, **1972**, *56*, 2257.
16. Hariharan, P. C.; Pople, J. A. "The Influence of Polarization Functions on Molecular Orbital Hydrogenation Energies", *Theor. Chim. Acta*, **1973**, *28*, 213.
17. Francl, M. M.; Pietro, W. J.; Hehre, W. J.; Binkley, J. S.; Gordon, M. S.; DeFrees, D. J.; Pople, J. A. "Self-Consistent Molecular Orbital Methods. XXIII. A Polarization-Type Basis Set for Second-Row Elements", *J. Chem. Phys.*, **1982**, *77*, 3654.
